# Serine/threonine kinase TBK1 promotes cholangiocarcinoma progression via direct regulation of β-catenin

**DOI:** 10.1038/s41388-023-02651-4

**Published:** 2023-03-16

**Authors:** Chong-Qing Gao, Zhen-Zhen Chu, Di Zhang, Yang Xiao, Xing-Yan Zhou, Jun-Ru Wu, Hui Yuan, Yu-Chuan Jiang, Dong Chen, Ji-Chun Zhang, Nan Yao, Kai-Yun Chen, Jian Hong

**Affiliations:** 1 Department of Pathophysiology, School of Medicine, Jinan University, Guangzhou, Guangdong 510630 China; 2 Department of Hepatological Surgery, the First Affiliated Hospital, Jinan University, Guangzhou, Guangdong 510632 China; 3 School of Medicine, Jinan University, Guangzhou, Guangdong 510632 China; 4 Department of Gastroenterology, Huizhou Municipal Central Hospital, Huizhou, 516001 Guangdong China; 5 Center of Hepato-Pancreato-Biliary Surgery, the First Affiliated Hospital, Sun Yat-sen University, Guangzhou, 510080 China; 6 Department of Physiology, School of Medicine, Jinan University, Guangzhou, 510632 China; 7 Department of General Surgery, Guangzhou Hospital Of Integrated Traditional And West Medicine, Guangzhou, Guangdong 510632 China

**Keywords:** Targeted therapies, Proteomics, Prognostic markers

## Abstract

Cholangiocarcinoma (CCA) is a highly heterogeneous and metastatic malignancy with a poor prognosis even after curative hepatectomy. Studies exploring its pathogenesis and identifying effective therapeutic targets are urgently needed. In this study, we found that TANK-binding kinase 1 (TBK1), a serine/threonine-protein kinase, showed a dynamic increase during the different stages of murine spontaneous CCA carcinogenesis (hyperplasia, dysplasia, and CCA). TBK1 was upregulated in human tissues, including intrahepatic (*n* = 182) and extrahepatic (*n* = 40) CCA tissues, compared with nontumor tissues, and the elevated expression of TBK1 was positively correlated with larger tumour diameter, lymph node metastasis, and advanced TNM stage. Functional studies indicated that TBK1 promoted CCA growth and metastasis both in vitro and in vivo. TBK1 directly interacts with β-catenin, promoting its phosphorylation at the S552 site and its nuclear translocation, which further activates EMT-related transcriptional reprogramming. GSK-8612, a TBK1 inhibitor or a kinase-inactivating mutation, effectively suppresses the above processes. In addition, we found that low-density lipoprotein receptor (LDLR), which mediates the endocytosis of cholesterol, was upregulated in CCA. Therefore, we designed a cholesterol-conjugated DNA/RNA heteroduplex oligonucleotide targeting TBK1 (Cho-TBK1-HDO), which could accumulate in CCA cells via LDLR, reduce the TBK1 mRNA level and inhibit intrahepatic metastasis of CCA. Besides, in the experimental group of 182 ICC patients, high TBK1 expression combined with high nuclear β-catenin expression predicted a worse prognosis. In summary, TBK1 might serve as a potential prognostic biomarker and therapeutic target for patients with CCA.

## Introduction

Cholangiocarcinoma (CCA), the second most common primary cancer of the liver, is a highly malignant epithelial neoplasm with cholangiocyte differentiation [1–3]. According to anatomical location, CCAs are classified as intrahepatic cholangiocarcinoma (ICC), perihilar CCA, or distal CCA, with the latter two collectively referred to as extrahepatic cholangiocarcinoma (ECC) [4]. In the past decade, there has been an increasing incidence (mainly of the intrahepatic subgroup) worldwide [5]. Although radical resection and systemic chemotherapy have shown remarkable improvements [6, 7], the prognosis of patients with CCA remains dismal due to malignant proliferation and metastasis [8, 9]. The development of targeted therapies that address disease pathogenesis or progression has lagged because of the complex and unclear pathogenesis [10]. Therefore, there is an urgent need to investigate the molecular mechanisms underlying CCA metastasis to develop potential therapeutic strategies to target CCA metastasis.

Epithelial-mesenchymal transition (EMT) plays a vital role in the induction of tumour cell invasion and metastasis [11]. EMT has been reported to promote tumour progression and metastasis in pancreatic ductal adenocarcinoma (PDAC) [6, 11]. CCA shares anatomic, embryologic, and genetic features with PDAC [12, 13]. However, the functions of EMT in CCA have not been studied thoroughly in vivo. EMT involves a cellular reprogramming process that drives epithelial cells into a mesenchymal-like phenotype and is characterized by the loss of epithelial surface markers such as E-cadherin and the acquisition of the mesenchymal markers vimentin and N-cadherin [14, 15]. Thus, EMT has been recognized as a prometastatic cellular event that promotes tumour cell invasion and malignant tumour progression [16, 17]. Recent studies have shown that a series of transcription factors are direct repressors of E-cadherin, including β-catenin, a dual-function protein implicated in transcriptional regulation and cell–cell adhesion [18]. In normal epithelial cells, most β-catenin proteins form adhesion complexes with E-cadherin and are located in cell‒cell adherens junctions at the membrane. In tumour cells, β-catenin detaches from the complex, and activation by nuclear translocation promotes the EMT process, further repressing E-cadherin expression [19]. Nevertheless, the molecular mechanisms by which β-catenin translocates into the nucleus remain largely unknown.

Tumour necrosis factor (TNF) receptor-associated factor (TRAF) family member-associated NF-κB activator TANK-binding kinase 1 (TBK1), which is also known as *NAK*, *T2K*, is a serine/threonine-protein kinase that plays essential roles in cancer development and progression [20, 21]. TBK1 has been demonstrated to contribute to tumorigenicity in human pancreatic cancer by promoting cell growth, migration, and invasion [22, 23]. Despite the growing interest in studying the roles and regulation of TBK1 in cancer, the precise molecular mechanisms governing TBK1 signalling and its subsequent impact on cancer biology remain incompletely understood.

This study found that TBK1 is upregulated in CCA and correlated with CCA metastasis. Enforced expression of TBK1 stimulated the metastatic potential of CCA cells via induction of the EMT process. In addition, we revealed that TBK1 directly interacts with and activates β-catenin and promotes its nuclear translocation. Moreover, we showed a positive correlation between TBK1 and the nuclear expression of β-catenin. Inhibition of TBK1 by GSK-8612 and Cho-TBK1-HDO effectively suppressed CCA intrahepatic metastasis. Taken together, our data indicate that the overexpression of TBK1 promotes CCA metastasis through β-catenin-mediated induction of EMT and that TBK1 has the potential to be a prognostic factor and therapeutic target of CCA.

## Results

### TBK1 is upregulated in CCA and correlates with aggressive clinicopathological characteristics

**Fig. 1:**
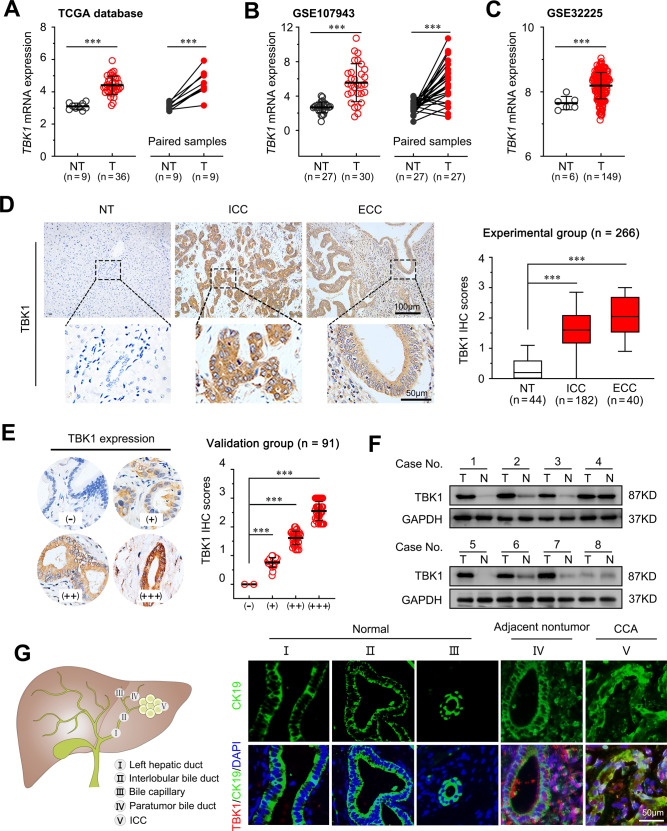
TBK1 is upregulated in CCA. The mRNA level of TBK1 among adjacent nontumor and CCA tissues (**A**) data from the TCGA, (**B**) GSE107943 (**C**), and GSE32225. **D** Representative images of IHC staining for TBK1 in the experimental groups, including normal hepatic (*n* = 44), CCA (*n* = 182), and ECC (*n* = 40). The scale bars are 100 μm (upper) and 50 μm (lower). **E** Representative images of IHC staining for TBK1 in the validation group (OUTDO Biotech, Shanghai, China, *n* = 91), including negative (−, 2.2%); weakly positive (+, 25.3%); moderately positive (++, 37.4%) and strongly positive (+++, 35.1%). **F** Western blot analysis of TBK1 expression in CCA tumour and paired adjacent nontumor tissues. T, tumour tissue, N, peritumoral normal tissues. **G** Representative immunofluorescence images of CK19 (green) and TBK1 (red) labelling in the same patient’s normal (I, left hepatic duct; II, interlobular bile duct; III, capillary bile duct), adjacent nontumor (IV, adjacent paratumor bile duct), and CCA tissues (V, ICC bile duct). Scale bar, 50 μm. ***P < 0.001.

To clarify the underlying role of TBK1 in CCA, the Tumour Immune Estimation Resource was used to analyse the transcriptome sequencing data from the TCGA dataset. The results revealed that TBK1 expression was upregulated in nine types of cancer, including CCA (Fig. 1A, and Supplementary Fig. 1A), suggesting that TBK1 may participate in the tumorigenesis and progression of several solid tumours. The gene set enrichment (GSE) dataset confirmed the trend of upregulation of TBK1 in CCA (Fig. 1B, C). Next, we examined the TBK1 expression status in hepatic tissues obtained from experimental group patients. IHC analysis showed a high level of cytosol-localized TBK1 in 76.4% (139/182) of the ICC and 85.0% (34/40) of the ECC compared to the nontumor tissues (*P* < 0.001; Fig. 1D, and Supplementary Fig. 1B, C). In addition, the upregulation of TBK1 in CCA was confirmed by a validation group (tissue microarray, *n* = 91, Outdo Biotech, Shanghai, China) (*P* < 0.001; Fig. 1E). Similar results were also observed in the immunoblot analyses (Fig. 1F). In addition, the expression of TBK1 was significantly higher in several CCA cell lines than in human normal bile duct HIBEpiC cells (Supplementary Fig. 1D). Furthermore, we examined the expression levels of TBK1 by immunofluorescence staining. Our results showed that TBK1 expression was higher in the bile ducts with CCA and lower in the adjacent bile ducts with tumour invasion, and no TBK1 expression was detected in adjacent nontumor tissues (including the left hepatic duct, interlobular bile duct, and capillary bile duct) (Fig. 1G and Supplementary Fig. 1E).

To further investigate the clinical significance of TBK1 expression in CCA, all 182 CCA patients with detailed clinical prognosis information were divided into two groups based on the overall expression level of TBK1: the high TBK1 expression group (*n* = 139) and the low TBK1 expression group (*n* = 43). As shown in Supplementary Table 1, the upregulation of TBK1 was significantly correlated with several aggressive clinicopathological characteristics, such as larger tumour diameter (*P* = 0.009), lymph node metastasis (*P* < 0.001), and advanced TNM stage (*P* = 0.001). The correlation between TBK1 and tumour size, lymph node metastasis, or TNM stage suggested that TBK1 may be involved in tumour progression in CCA.

### TBK1 is upregulated in both rat and mouse spontaneous CCA model tissues

**Fig. 2:**
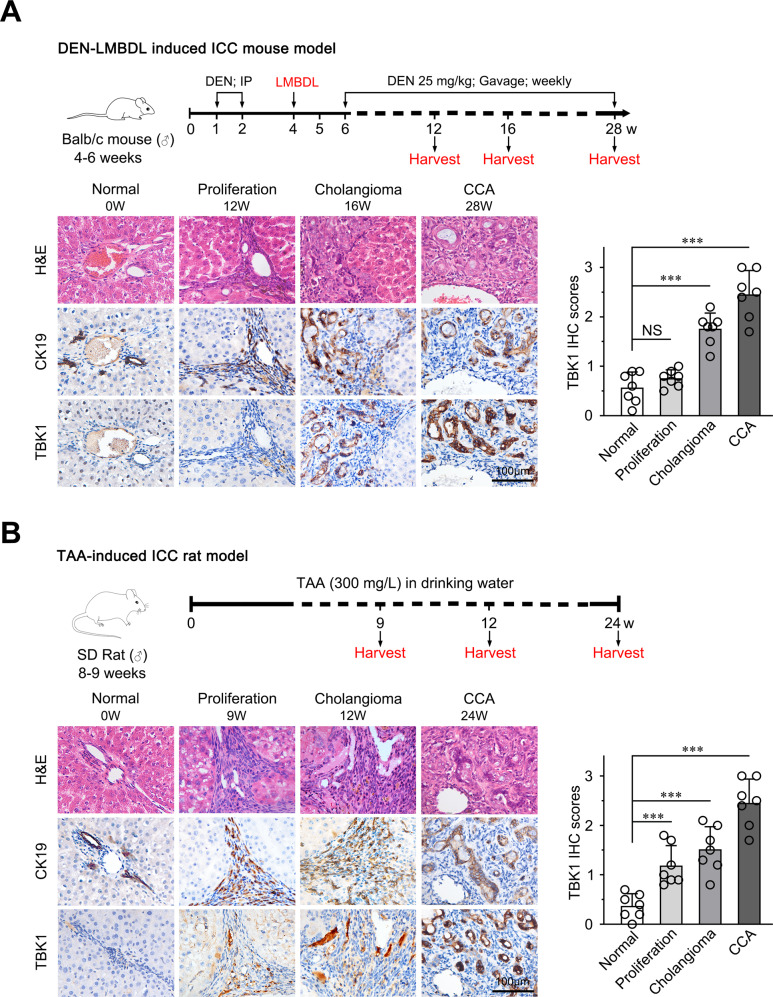
TBK1 is upregulated in rat and mouse spontaneous induction models. **A** Schematic of the experimental design of the DEN/LMBDL mouse CCA model and representative images of H&E, CK19, and TBK1 staining in the different stages of the mouse CCA models. Scale bar, 100 μm. **B** Schematic of the experimental design of the TAA rat CCA model and representative images of H&E, CK19, and TBK1 staining in the different stages of the rat CCA models. Scale bar, 100 μm. NS, not significant, **P < 0.01, ***P < 0.001.

To further verify the underlying role of TBK1 in CCA development and progression, we used two previously established murine spontaneous induction CCA models, including a diethylnitrosamine/left median bile duct ligation (DEN/LMBDL) mouse CCA model and a thioacetamide (TAA) rat CCA model. Next, TBK1 expression was evaluated in spontaneous CCA samples. We found that TBK1 expression was dynamically upregulated during the different stages of CCA carcinogenesis (hyperplasia, dysplasia, and CCA) in spontaneous CCA models (Fig. 2A, B). These data collectively demonstrated that the upregulation of TBK1 is a frequent event in CCA.

### Upregulation of TBK1 promotes CCA cell growth, motility, and metastasis both in vitro and in vivo

**Fig. 3:**
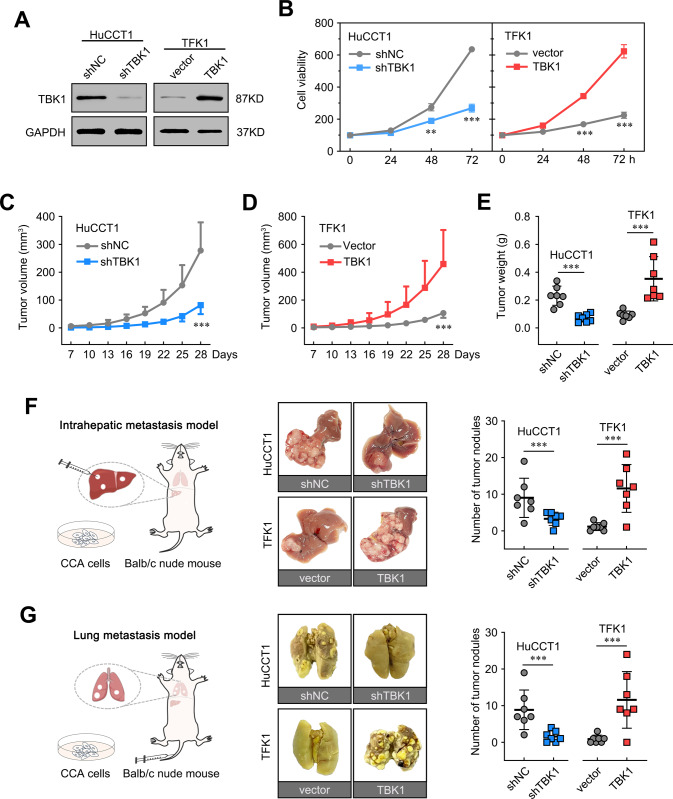
TBK1 promotes CCA cell growth, migration, and invasion both in vitro and in vivo. **A** Knockdown of TBK1 in HuCCT1 cells and overexpression of TBK1 in TFK1 cells, as confirmed by immunoblot analysis. **B** Effects of stable TBK1 depletion and overexpression on proliferation using the Cell Counting Kit-8 assay. **C**, **D** TBK1 stable depletion and overexpression effects on the growth of in vivo subcutaneous xenograft tumours. Tumour size was monitored every three days, and tumour volume was calculated as follows: length × width² × 0.5. Tumour weight was recorded following harvesting. The results are presented as the means ± SDs. **E** The weight of subcutaneous tumours by injection of the indicated cells. **F** HuCCT1 and TFK1 cells infected with lentiviruses, as previously described, were inoculated orthotopically into the livers of 6-week-old male Balb/c nude mice (left). Representative gross images (middle) and quantification of liver metastasis nodules (right). The results are presented as the means ± SDs (*n* = 7 per group). **G** HuCCT1 and TFK1 cells infected with lentiviruses, as previously described, were injected into mouse tail veins of 6-week-old male Balb/c nude mice (left) to construct a lung metastasis model representative gross image (middle), and the quantification of lung metastasis nodules (right). The results are presented as the means ± SDs. (*n* = 7 per group). ***P < 0.001.

To elucidate the functions of TBK1 in CCA progression, we knocked down TBK1 in HuCCT1 cells, which exhibited relatively high endogenous TBK1 levels. In addition, we overexpressed TBK1 in TFK1 cells, which exhibited relatively low endogenous TBK1 levels (Fig. 3A). The CCK8 assay results showed that the overexpression of TBK1 increased the proliferation ability of CCA cells (Fig. 3B). Wound healing migration assays, Transwell migration assays, and Matrigel invasion assays revealed that the overexpression of TBK1 enhanced the growth migration and invasion ability. In contrast, the knockdown of TBK1 expression significantly attenuated these effects (Supplementary Fig. 2A, B). Similarly, the knockdown of TBK1 by specific siRNAs repressed cell growth, migration, and invasion in HuCCT1 and RBE cells highly expressing TBK1 (Supplementary Fig. 3A–D).

Subsequently, a mouse subcutaneous xenograft model was developed to evaluate the effect of TBK1 on CCA progression in vivo. The tumour growth curve and tumour weight results showed that tumours from TBK1-overexpressing cells grew significantly faster (Fig. 3C–E). Subsequently, we assessed Ki-67 expression by immunohistochemistry and found more positive cells in xenografts derived from TBK1-overexpressing cells than in those derived from control cells (Supplementary Fig. 4A). These results were further validated in human CCA tissues (Supplementary Fig. 4B). Furthermore, an orthotopic CCA model was established to determine whether TBK1 had the same effect on CCA metastasis in vivo. As shown in Fig. 3F, depletion of TBK1 significantly inhibited liver colonization of HuCCT1 cells, whereas overexpression of TBK1 significantly promoted liver colonization of TFK1 cells. Next, the cells mentioned above were used to establish a lung metastasis mouse model by tail vein injection. More metastatic nodules were observed in mice injected with TBK1-overexpressing cells than in those injected with control cells (Fig. 3G). These results indicate that TBK1 can promote the growth, invasion, and metastasis of CCA cells both in vitro and in vivo.

### Upregulation of TBK1 promotes EMT in CCA cells

**Fig. 4:**
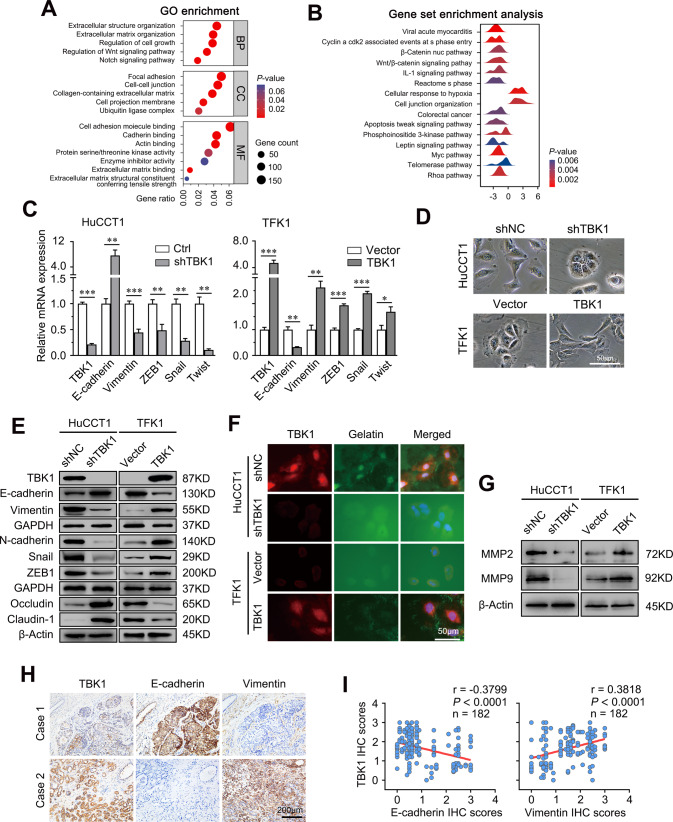
Upregulation of TBK1 promotes EMT in CCA. **A** GO enrichment analysis of the DEGs. **B** Ridgeline plot depicting the significantly enriched signalling pathways of the DEGs revealed by GSEA. **C** Fold change in the mRNA levels of TBK1, E-cadherin, vimentin, *ZEB1*, *Snail*, and *Twist* in cells with stable TBK1 depletion and overexpression. The data are the means ± SDs and represent three independent experiments. **D** The cells morphology in cells with stable TBK1 depletion and overexpression Scale bar = 50 μm. **E** The protein levels of E-cadherin, N-cadherin, vimentin, *Snail*, *ZEB1*, occludin, and claudin-1 in cells with stable TBK1 depletion and overexpression was evaluated by Western blotting. **F** FITC labeled gelatin (green) and TBK1 (red) staining was performed to measure the gel degrading ability of the cells. Scale bar = 50 μm. **G** The protein levels of MMP2 and MMP9 in cells with stable TBK1 depletion and overexpression was evaluated by Western blotting. **H** Representative immunohistochemical staining of TBK1, E-cadherin, and vimentin in human CCA tissues. Scale bar = 200 μm. **I** Correlation analysis between TBK1 and E-cadherin and vimentin expression in liver tumour tissues from patients with CCA (*n* = 182). **P < 0.01, ***P < 0.001.

To further explore the potential mechanism by which TBK1 promotes tumour progression, we performed RNA sequencing, and the results showed approximately 3101 differentially expressed genes (DEGs) after TBK1 knockdown (Supplementary Fig. 5A). The gene rank of these differentially expressed genes indicated that epithelial phenotype-associated genes were significantly upregulated, while mesenchymal phenotype-associated genes were downregulated (Supplementary Fig. 5B). Moreover, Gene Ontology (GO) enrichment analysis and Gene Set Enrichment Analysis (GSEA) revealed that TBK1 knockdown regulated genes associated with several EMT-related signaling pathways, such as the Wnt signalling pathway and the β-catenin nuclear translocation pathway (Fig. 4A, B). These data suggested that TBK1 knockdown significantly affected EMT in CCA cells.

Next, the mRNA expression levels of E-cadherin and a series of EMT inducers, including vimentin, *ZEB1*, *Snail*, and *Twist1*, were measured in both TBK1-overexpressing and TBK1 knockdown cells. Overexpression of TBK1 stimulated the EMT process (Fig. 4C). RNA-seq data shows transcription factors that positively regulate EMT process such as *ZEB1*, *SNAI2*, *MYC*, *SMAD5*, and *STST1*, were significantly downregulated following the TBK1 knockdown while negatively regulating the EMT process show the opposite results (Supplementary Fig 6). TBK1-positive CCA cells were loosely packed and polarized with morphologies reminiscent of spindle-shaped stromal or mesenchymal cells (Fig. 4D). TBK1 promote EMT process was further demonstrated by down-regulation of epithelial markers, such as E-cadherin, and up-regulation of mesenchymal markers, such as N-cadherin, vimentin, and EMT transcriptional factors, including *Snail* and *ZEB1*. (Fig. 4E). The absence of tight junction proteins and the dissolution of the extracellular matrix are early events in EMT [24]. Consistently, the overexpression of TBK1 attenuated the expression of tight junctions protein occludin and claudin-1 (Fig. 4E), meanwhile enhanced the expression of MMP-2 and MMP-9 (Fig. 4G), which promotes the degrading of extracellular matrix (Fig. 4F). We then assessed the relationship between TBK1 and EMT in clinical CCA tissues. Immunohistochemical staining showed that CCA patients with low TBK1 expression had higher E-cadherin and lower vimentin expression levels than CCA patients with high TBK1 expression (Fig. 4H). Moreover, correlation analyses showed that the expression of TBK1 correlated with the expression of E-cadherin (r = −0.3799, *P* < 0.001) and vimentin (r = 0.3818, *P* < 0.001) (Fig. 4I), suggesting that TBK1 is highly expressed mainly in mesenchymal phenotype cholangiocarcinoma tissues. Taken together, these results demonstrated that TBK1 plays a vital role in the early events of CCA metastasis by promoting mesenchymal phenotype.

### TBK1 promotes the EMT process through β-catenin activation

**Fig. 5:**
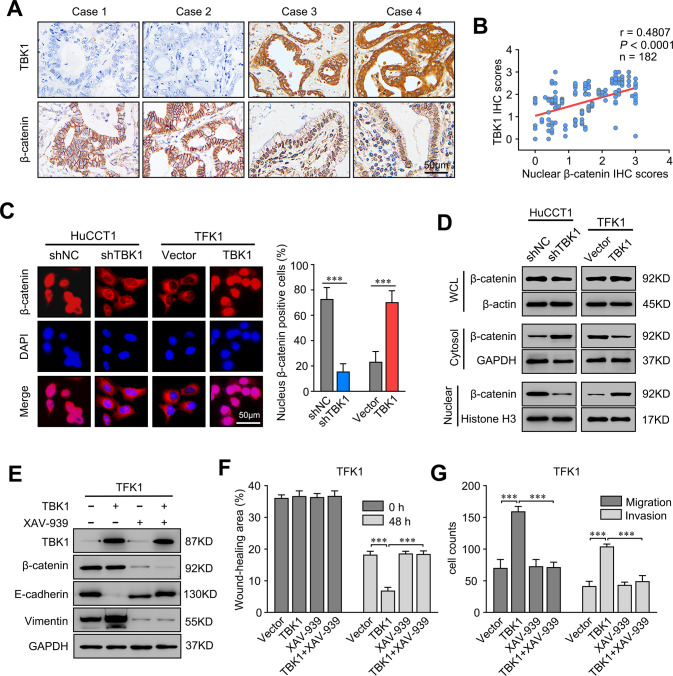
TBK1 promotes the EMT process through β-catenin activation. **A** Representative immunohistochemical staining of TBK1 and β-catenin in human CCA tissues. The scale bars are 100 μm. **B** Correlation analysis between TBK1 and nuclear β-catenin expression in CCA tumour tissues from 182 patients. **C** β-Catenin expression in the indicated cells, as detected by an immunofluorescence assay. The merged images show overlays of β-catenin (red) and nuclear staining by DAPI (blue); scale bar: 50 μm. **D** β-Catenin expression in whole-cell lysates and the cytoplasmic and nuclear fractions, as detected by immunoblot analysis. **E** Relative expression levels of TBK1, E-cadherin, vimentin, and β-catenin in the indicated cells treated with or without XAV-939, a potent tankyrase inhibitor that inhibited nuclear entry of β-catenin. **F** Wound healing migration assays were performed with the indicated CCA cells treated with or without XAV-939. The data are the means ± SDs and represent three independent experiments. **G** Transwell migration and Matrigel invasion assays performed with the indicated CCA cells treated with or without XAV-939. The data are the means ± SDs and represent three independent experiments. ***P < 0.001.

β-Catenin plays a critical role in the induction of EMT during CCA metastasis [25]. To determine whether β-catenin is involved in the TBK1-mediated EMT process, we assessed the clinical relationship between TBK1 and β-catenin in CCA tissues. IHC showed that CCA patients with low TBK1 expression displayed lower nuclear β-catenin expression levels than CCA patients with high TBK1 expression (Fig. 5A). Consistent with this finding, correlation analyses showed that the protein expression level of TBK1 was closely associated with that of nuclear β-catenin (r = 0.4807, *P* < 0.001) (Fig. 5B). These results suggested that TBK1 might promote the nuclear translocation of β-catenin.

Similar to the above findings, β-catenin was localized primarily in the cytoplasm of cells with low TBK1 expression. However, nuclear β-catenin expression was significantly enhanced in cells with high TBK1 expression (Fig. 5C). In addition, the cytosolic and nuclear fractions of cell lysates were separated to verify this finding. Neither overexpression nor knockdown of TBK1 significantly affected the total β-catenin level. However, TBK1 overexpression decreased the cytosolic β-catenin level and increased the nuclear β-catenin level, while knockdown of TBK1 exerted the opposite effect (Fig. 5D).

To determine whether the activation of β-catenin is essential for the TBK1-mediated promotion of EMT, XAV939 [26, 27] was used to inhibit nuclear entry of β-catenin in TBK1-overexpressing cells. The results showed that inhibition of β-catenin nuclear entry blocked the TBK1-mediated upregulation of vimentin and the downregulation of E-cadherin (Fig. 5E). In line with this finding, the EMT-mediated upregulation of cell invasion and migration was abolished by β-catenin inhibition (Fig. 5F, G). These results demonstrated that TBK1 overexpression leads to β-catenin activation, promoting the EMT process.

### TBK1 promotes β-catenin nuclear translocation by phosphorylation at S552

**Fig. 6:**
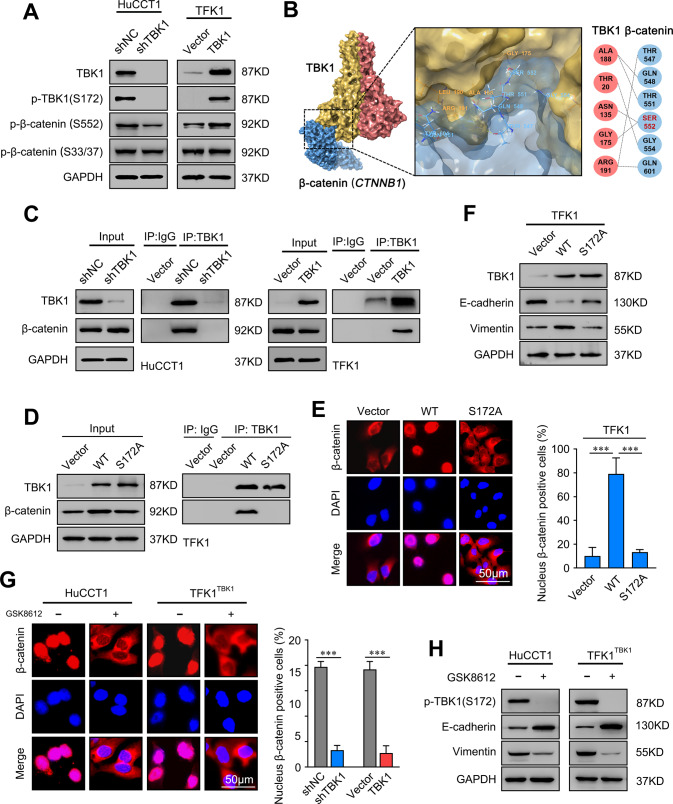
TBK1 promotes β-catenin nuclear translocation by phosphorylation at S552. **A** Western blot analysis of the expression of the TBK1, p-TBK1, p-β-catenin (S552), and p-β-catenin (S33/37) proteins in the indicated CCA cells. **B** Molecular docking model of TBK1 with β-catenin (*CTNNB1*)**. C** Immunoprecipitation of TBK1 with β-catenin in the indicated CCA cells, as detected by immunoblot analysis. **D** 293 T cells transfected with tagged vector, wild-type TBK1 (WT), or serine-mutated TBK1 (S172A) were immunoprecipitated with an anti-TBK1 antibody. The β-catenin and TBK1 levels are indicated. **E** β-Catenin expression in the indicated cells, as detected by immunofluorescence assay. The merged images show overlays of β-catenin (red) and nuclear staining by DAPI (blue). Scale bar = 50 μm. **F** Relative expression levels of TBK1, E-cadherin, and vimentin in the indicated cells. **G** β-Catenin expression in the indicated cells treated with or without GSK-8612 (2.0 μM), as detected by immunofluorescence assay. The merged images show overlays of β-catenin (red) and nuclear staining by DAPI (blue). Scale bar = 50 μm. **H** Relative expression levels of p-TBK1, E-cadherin, and vimentin in the indicated cells treated with or without GSK-8612 (2.0 μM). ***P < 0.001.

The phosphorylation of β-catenin at S552 has been demonstrated to promote β-catenin activation and nuclear translocation [28]. Various kinases are activated, leading to the phosphorylation and activation of transcription factors. Thus, we hypothesize that TBK1, a serine/threonine-protein kinase, stimulates β-catenin activation. To explore the mechanism by which TBK1 promotes β-catenin activation, we first examined the specific S552 phosphorylation of β-catenin by immunoblot analyses. The levels of S552 phosphorylation were significantly reduced in HuCCT1-shTBK1 cells, and the opposite results were obtained in TBK1-overexpressing TFK1 cells, suggesting that TBK1 contributes to β-catenin activation via phosphorylation of S552 (Fig. 6A). The experimental findings were confirmed by molecular simulations (Fig. 6B). The interactions were further validated by immunoprecipitation with a TBK1 antibody (Fig. 6C). Altogether, these findings revealed that TBK1 overexpression stimulates the nuclear translocation and activation of β-catenin through direct interaction.

As shown in Fig. 6D, E, we constructed a TBK1 mutant in S172 and found that the mutation eliminated the interaction between TBK1 and β-catenin and the nuclear translocation of β-catenin. Similarly, the TBK1-S172A mutant did not induce the EMT process (Fig. 6F).

We further confirmed that GSK-8612 (2.0 μM), a TBK1 S172-specific inhibitor (Supplementary Fig. 7A), did not affect proliferation but effectively inhibited TBK1 phosphorylation at S172 (Supplementary Fig. 7B, C). Immunofluorescence analysis showed that GSK-8612 notably inhibited β-catenin nuclear expression in the indicated cells (Fig. 6G). Moreover, increased E-cadherin expression and decreased vimentin expression were observed in both HuCCT1 and TFK1 cells (Fig. 6H). Taken together, these results demonstrated that the active site of TBK1 is crucial for TBK1-mediated β-catenin activation.

### Cho-TBK1-HDO treatment and pharmacological inhibition of TBK1 reduce CCA cell growth both in vitro and in vivo

**Fig. 7:**
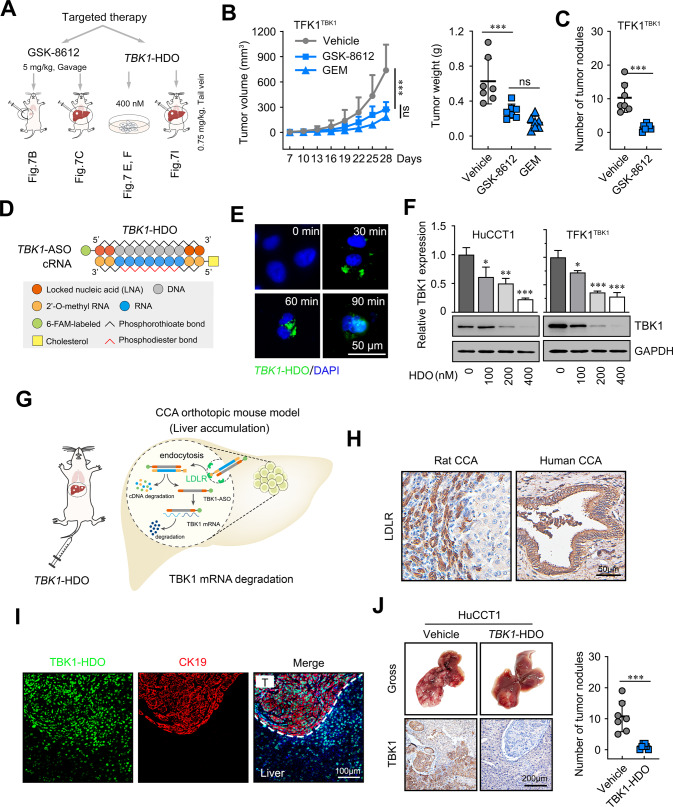
Cho-TBK1-HDO treatment and pharmacological inhibition of TBK1 reduce CCA cell growth both in vitro and in vivo. **A** Schematic flow chart of TBK1 targeted therapy. **B** The effects of different treatments on the growth of in vivo subcutaneous xenograft tumours. Male BALB/c nude mice were injected subcutaneously with 2 × 10^6^ HuCCT1 cells, and tumours were allowed to grow for 7 days before being randomized for starting the indicated treatments (*n* = 7 per group). Tumour size was monitored every three days, and tumour volume was calculated as follows: length × width² × 0.5. Tumour weight was recorded following harvesting. The results are presented as the means ± SDs. GEM, gemcitabine. **C** Statistical analysis of tumour nodules in mouse liver orthotopic xenograft tumours. **D** Schematic illustration of the construction of Cho-TBK1-HDO. **E** HuCCT1 cells were treated with Cho-TBK1-HDO after 30 min, 60 min, and 90 min by immunofluorescence assay. Merged images represent overlays of Cho-TBK1-HDO (green) and nuclear staining by DAPI (blue). Scale bar = 50 μm. **F** Dose-dependent reduction of gene silencing in HuCCT1 and TFK1-TBK1 cells with Cho-TBK1-HDO. Data are representative of at least three independent experiments. **G** After Cho-TBK1-HDO tail vein injection, the Cho-TBK1-HDO, labelled with cholesterol, accumulated in hepatocytes by hepatocyte surface receptor LDLR-mediated endocytosis and was more effective at reducing the expression of TBK1 mRNA in the liver. **H** Representative images of IHC staining for LDLR in rat and human CCA tissues. The scale bars are 50 μm. **I** Fluorescence micrographs of mouse liver tissues after treatment with Cho-TBK1-HDO. Green: 6-FAM-labelled DNA/LNA gapmer; Red: CK19; Blue: DAPI. Scale bar = 100 μm. **J** Representative images of tumour morphology of liver tissue and IHC in vivo at the endpoint and statistical analysis of tumour nodules. *P < 0.05, **P < 0.01, ***P < 0.001.

Given that depletion of TBK1 by shRNA, siRNA, and TBK1 inhibitors inhibited human CCA cell growth in vitro, we further evaluated the effect of TBK1 inhibitors in vivo, including repressing TBK1 kinase activity by GSK-8612 and inhibiting protein expression by HDO (Fig. 7A). We first established subcutaneous xenograft and orthotopic xenograft tumour models in male nude mice. After successful model construction, we treated mice with the TBK1 kinase inhibitor GSK-8612 or vehicle for 3 weeks by oral gavage (5 mg/kg once daily). The results showed that GSK-8612 significantly inhibited subcutaneous xenograft and growth, which showed the same effect as gemcitabine’ (GEM), the first-line treatment for CCA. (Fig. 7B, and Supplementary Fig. 8A, B). The effects of drug treatments in vivo were further confirmed in an orthotopic transplantation model (Fig. 7C, and Supplementary Fig. 8C).

Recently, antisense oligonucleotides (ASO) targeting as a therapeutic approach has been applied in many diseases, including cancer [29, 30]. To date, 10 ASO drugs have been commercially approved for the treatment of human disease [31]. Here, we designed a cholesterol-conjugated DNA/RNA heteroduplex oligonucleotide targeting TBK1 (Cho-TBK1-HDO), to enhance the activity of the ASO (Fig. 7D). For in vitro studies, the Cho-TBK1-HDO significantly decreased TBK1 mRNA and protein expression in the indicated cells in both a time- and dose-dependent manner (Fig. 7E, F). Notably, cholesterol is metabolized primarily in the liver, where it enters the cell mainly via low-density lipoprotein receptor (LDLR)-mediated endocytosis in the form of cholesterol ester [32–34]. The Cho-TBK1-HDO, labelled with cholesterol, could accumulate in hepatocytes and CCA cells via LDLR and reduce the expression of TBK1 mRNA in the liver (Fig. 7G). A relatively selective accumulation of fluorescence-labelled Cho-TBK1-HDO in the hepatocytes was detected by histological analysis after Cho-TBK1-HDO tail vein injection (Supplementary Fig. 9A). The expression level of LDLR in HuCCT1 cells was comparable to that in LO2 hepatocytes (Supplementary Fig. 9B). Consistent with the above observations, LDLR was upregulated in the rat spontaneous model and human CCA tissues (Fig. 7H). Therefore, we hypothesized that Cho-TBK1-HDO could accumulate in cholangiocarcinoma tissue by LDLR and play a tumour suppressor role. Then, we established an orthotopic CCA model by HuCCT1 (high LDLR expression) cells, and the results of the immunofluorescence assay showed a relatively selective accumulation of fluorescence-labelled Cho-TBK1-HDO in the CCA cells (Fig. 7I). Moreover, Cho-TBK1-HDO significantly reduced the number of orthotopic xenograft tumours (Fig. 7J). The results further suggested that Cho-TBK1-HDO may be a novel nucleic acid therapeutic for cholangiocarcinoma. Taken together, these findings indicate that pharmacological inhibition of TBK1 decreases CCA cell growth both in vitro and in vivo.

### Upregulation of TBK1 predicts poor survival in CCA patients

**Fig. 8:**
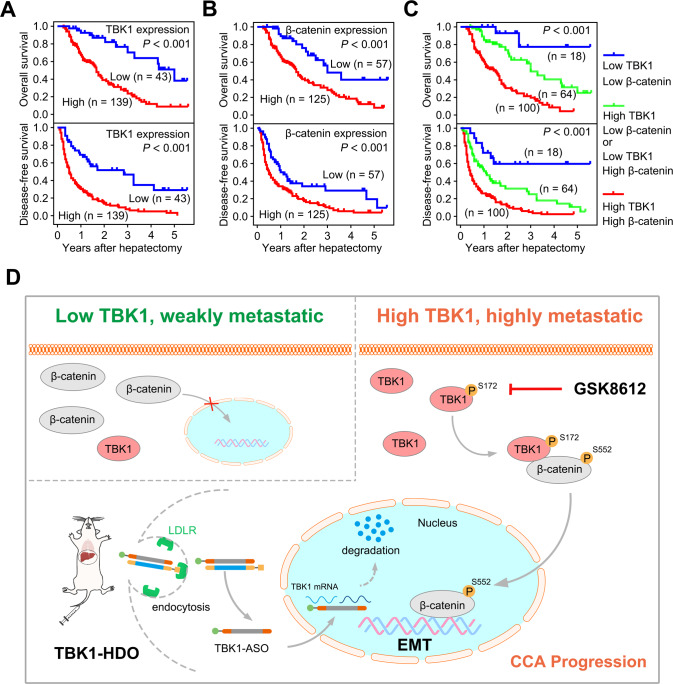
Upregulation of TBK1 predicts poor survival in CCA patients. **A** Overall survival (OS) and disease-free survival (DFS) curves of patients from the First Affiliated Hospital, Sun Yat-sen University dataset (our cohort, *n* = 182) with high or low TBK1 expression levels. **B** Overall survival (OS) and disease-free survival (DFS) curves of patients from the First Affiliated Hospital, Sun Yat-sen University dataset (our cohort, *n* = 182) with high or low β-catenin expression levels. **C** Patients were stratified by TBK1 and nuclear β-catenin expression levels. CCA patients were classified into three groups according to TBK1 and nuclear β-catenin expression: Group 1 (*n* = 18): low TBK1 and β-catenin expression; Group 2 (*n* = 64): high TBK1 and low β-catenin expression or low TBK1 and high β-catenin expression; and Group 3 (*n* = 100): high TBK1 and β-catenin expression. **D** The proposed model of TBK1 inhibition of CCA progression.

The prognostic implication of TBK1 in CCA was explored next. Kaplan–Meier survival analysis revealed that patients with a high level of TBK1 expression exhibited significantly poorer overall survival (hazard ratio (HR), 3.78; 95% confidence interval (CI), 2.48–5.75) and disease-free survival (hazard ratio (HR), 2.97; 95% confidence interval (CI), 2.11–4.16) in our cohorts (Fig. 8A). Similar results were observed in cohort 1 (Supplementary Fig. 10A). Unfortunately, it showed no significance in the TCGA, which might be related to the small sample size. (Supplementary Fig. 10B, C).

Consistently, nuclear β-catenin overexpression resulted in significantly poorer overall survival and disease-free survival (Fig. 8B). Interestingly, we found that patients with low expression of both TBK1 and nuclear β-catenin had the best prognosis; in contrast, patients with high expression of both TBK1 and nuclear β-catenin had the worst prognosis (Fig. 8C). Subsequently, the TBK1 expression status and prognostic clinicopathological parameters identified by univariate analysis (*P* < 0.01) were entered into a multivariate model to identify independent predictors of overall survival. We found that TBK1 upregulation was an independent statistically significant risk factor for overall survival (*P* < 0.001, Supplementary Fig. 10D), suggesting that upregulation of TBK1 may play a pivotal role in the overall survival of CCA patients. Taken together, these results indicated that the combination of TBK1 and nuclear β-catenin could serve as a biomarker in CCA for evaluating the metastatic potential and predicting the prognosis of CCA patients.

## Discussion

Local and systemic metastasis is a major contributor to the dismal prognosis of intrahepatic cholangiocarcinoma (CCA) [35, 36]. Evidence indicates that the β-catenin-mediated epithelial-mesenchymal transition (EMT) process plays a vital role in cancer metastasis but has been defined as an undruggable target [37, 38]. This study found that TBK1, a novel regulator upstream of β-catenin, activates β-catenin via protein‒protein interaction and then promotes EMT and CCA metastasis (Fig. 8D). In contrast, inhibiting the expression of TBK1 exerts the opposite effects. Moreover, we demonstrated that TBK1 plays a vital role in mediating the intrahepatic metastasis and lung colonization of CCA cells in mouse models.

TBK1 plays a vital role in cancer development and progression [39, 40]. Previous studies have confirmed the distinct role of TBK1 in certain types of cancers, including pancreatic cancer, hepatocellular carcinoma, and breast carcinoma [41]. Under hypoxic conditions, TBK1 was hyperactivated (increased pSer172) and stabilized, thereby promoting cancer cell growth [22]. Our previous study demonstrated that high TBK1 expression enhanced hepatocellular carcinoma invasion and predicted poor prognosis [21]. However, the role of TBK1 has not been reported in CCA [12]. In the present study, we found that TBK1 is highly expressed mainly in mesenchymal phenotype cholangiocarcinoma tissues, and exerts a critical role in maintaining the tumor-promoting function indicating that TBK1 is also a potential therapeutic target for CCA.

The integrative molecular analysis has reported on the mesenchymal molecular subtypes of CCA, provides a better understanding of the molecular landscape of this clinically-challenging neoplasm, and is helpful to identify novel therapeutic targets [42, 43]. Consistent with the studies reported by Montal et al. [42], the mesenchymal molecular subtypes related gene, such as *SPARC, CDH11, COMP, VIM*, and *COL5A1* were significantly downregulated following TBK1 knockdown (Supplementary Fig. 11A). Follow the bioinformatics methods in the above literature, we also found in GEO database (GSE132305 and GSE32225), TBK1 was highly expressed in cholangiocarcinoma with a mesenchymal phenotype (Supplementary Fig. 11B, C). TBK1 expression had a positive correlation with the hallmark pathways of mesenchymal subtype, such as, epithelial-mesenchymal transition signaling pathway (*P* = 0.00025, *R* = 0.03829) and the TNFA signaling via NFκB signaling pathway (*P* = 0.00019, *R* = 0.03895) in dataset GSE32225 (Supplementary Fig. 11D). As we know, TBK1 is an activator of the NFκB signaling pathway and NFκB is also known as a stimulator of the EMT process [44, 45]. These results further suppport that TBK1 may further promote cholangiocarcinoma progression through regulation of EMT.

In recent years, it has become increasingly clear that EMT plays an essential role in cancer metastasis [44]. However, the most recent advances in EMT research have questioned the requirement for EMT in tumour metastasis. In one study, primary pancreatic ductal adenocarcinoma (PDAC) mouse models showed that EMT suppression does not alter PDAC metastasis [45]. In our study, we demonstrated that TBK1 upregulation induced the β-catenin-mediated EMT process. Since we proved that TBK1 promoted the migration and invasion of CCA cells, we hypothesized that TBK1 might stimulate CCA metastasis by promoting EMT. To exclude the possibility that EMT is dispensable for TBK1-induced CCA metastasis, we utilized a specific inhibitor targeting β-catenin, a key transcription factor involved in EMT, and concluded that EMT was required for TBK1-induced CCA metastasis. β-Catenin, as a critical factor in the Wnt/β-catenin pathway, is an essential transcriptional coactivator regulating EMT in cancers. It has been widely reported that the Wnt/β-catenin pathway plays a crucial role in cholangiocarcinoma tumorigenesis and metastasis [46]. In addition, our evidence has indicated that β-catenin is overexpressed in CCA tissues (characterized by increased nuclear expression in cancer cells) and promotes the EMT of CCA cells. Together, these results further verified that TBK1 promotes EMT by increasing the nuclear expression of β-catenin.

To our knowledge, the present study describes a novel mechanism by which TBK1 promotes EMT in cancer. We demonstrated that TBK1 activated the Wnt/β-catenin pathway. Although TBK1 did not enhance the total expression levels of β-catenin, it promoted the nuclear translocation of β-catenin, thereby stimulating the transcription of β-catenin downstream genes. TBK1 is located in the cytoplasm and functions via protein‒protein interactions. Interestingly, β-catenin was also present in the cytoplasm, suggesting that TBK1 might promote the nuclear translocation of β-catenin through direct protein‒protein interactions. We confirmed the interaction between TBK1 and β-catenin by immunoprecipitation and molecular docking simulation to verify this hypothesis. Collectively, the results of our study linked TBK1 with β-catenin, thus clarifying the underlying mechanism of TBK1-mediated CCA metastasis. However, the detailed mechanisms by which the interaction between TBK1 and β-catenin promotes the nuclear translocation of β-catenin require further investigation.

Gemcitabine combined with cisplatin has long been a first-line treatment for patients with unresectable or metastatic CCA [47, 48]. This study found that the GSK-8612, a specific inhibitor for blocking TBK1 S172 phosphorylation, was identical to gemcitabine, significantly suppressed the growth, migration, and metastasis of CCA cells. Cholesterol-conjugated DNA/RNA heteroduplex oligonucleotides (HDO) were previously reported as a new nucleic acid drug class [32]. Here, we also designed a cholesterol-conjugated DNA/RNA heteroduplex oligonucleotide targeting TBK1 (Cho-TBK1-HDO), which could accumulate in CCA cells via LDLR. HDO technology improved the efficacy of mRNA-targeted therapeutic oligonucleotides [49]. The ligand-conjugated DNA/RNA heteroduplex reveals a novel class of oligonucleotide drugs for human gene therapy.

Our study demonstrated that TBK1 promotes the EMT process by binding to β-catenin and increasing its nuclear expression in CCA. Knockdown of TBK1 abrogated the tumour-promoting effect and inhibited CCA progression. Overexpression of TBK1 and β-catenin in CCA is a strong indicator of high tumour aggressiveness and correlates with poor clinical outcomes. In conclusion, we found that TBK1 plays a vital role in CCA metastasis and could be a potential prognostic biomarker and therapeutic target for CCA.

## Materials and methods

### Human samples

We analysed the features of CCA with a tissue microarray that included 91 patients (OUTDO Biotech, Shanghai, China) and a cohort including 182 ICC patients who had undergone curative liver resection at The First Affiliated Hospital, Sun Yat-sen University between January 2015 and December 2020. In addition, 44 normal hepatic tissues obtained from patients who underwent resection due to benign hepatic lesions were used as normal controls. Another 40 ECC specimens were collected from the First Affiliated Hospital of Jinan University.

The use of the clinical specimens for research purposes was approved by the Jinan University Ethics Committee and IEC for Clinical Research and Animal Trials of the First Affiliated Hospital of Sun Yat-Sen University (ethics approval number: [2021]678) and was performed in accordance with the Declaration of Helsinki. Written informed consent was obtained from each patient at each institution.

### Cell culture

Human intrahepatic biliary epithelial cells HIBEpiC and the CCA cell line QBC939 were purchased from Zhenke Biotechnology Co., Ltd. (Shenzhen, China). Human CCA cell lines (RBE, HuH28, TFK1, and Hccc9810) were purchased from Lvyuan Dade Biological Science and Technology Co., Ltd. (Beijing, China). The human CCA cell line HUCCT1 was purchased from Suyan Biotechnology Co., Ltd. (Guangzhou, China). Cells were identified by polymorphic short tandem repeat (STR) profiling and tested to exclude mycoplasma contamination every month. Cells used in this research were cultured in RPMI-1640 medium (Gibco, Gaithersburg, MD) supplemented with 10% foetal bovine serum and 1% penicillin-streptomycin in a humidified incubator at 37 °C and 5% CO_2_.

### Chemicals

For in vitro experiments, GSK-8612 (Selleck Chemicals, Shanghai, China) was dissolved in DMSO (Sigma‒Aldrich, St. Louis, MO) and further diluted to the required concentration. For in vivo experiments, a GSK-8612 suspension was prepared in 0.5% carboxymethyl cellulose sodium typical saline solution.

### The thioacetamide model of CCA

The thioacetamide (TAA) rat model of cholangiocarcinoma was established in our previous report [50]. TAA (Sigma) was given in drinking water at a standard dose of 0.03% for male Sprague–Dawley (SD) rats (350 ± 20 g). Foci of cholangiocyte proliferation could be observed in the 9th week, and dysplasia could be detected in the 12th week. Whitish and visible CCA tumours could be observed in the 16th week after administration. All animals developed multiple different-sized invasive tumours at 24 weeks.

### The diethylnitrosamine/left median bile duct ligation (DEN/LMBDL) model of CCA

The construction of this model was performed as in our previous report [50]. To achieve tumour development in mice, we subjected 7-week-old male BALB/c mice to two separate weekly intraperitoneal injections of 100 mg/kg diethylnitrosamine (DEN, Sigma). After 2 weeks, left median bile duct ligation was performed in all experimental mice. After one week, DEN (25 mg/kg) was administered by oral gavage once a week for a total of 28 weeks, and this led to CCA formation.

### Cell proliferation assay

The cell proliferation ability was determined using the Cell Counting Kit-8 (Dojindo, Kumamoto, Japan). Briefly, 3000 cells were seeded into 96-well plates (Corning). Each well medium was replaced with an equal volume of fresh medium containing 10% CCK-8 reagent at the indicated times. After incubating cells at 37 °C for 2 h, absorbance was measured using an enzyme-linked immunosorbent instrument (Thermo Fisher Scientific, Waltham, MA) at 450 nm.

### Transwell migration and Matrigel invasion assays

Both assays used a transwell membrane (8 μm pore size, 6.5 mm diameter; Corning Costar). A total of 2.5 × 10^4^ cells were plated in the top chambers for the transwell migration assay. The top chambers were filled with serum-free medium, and the bottom chambers were filled with migration-inducing medium (with 10% FBS). The filters were fixed with 4% paraformaldehyde after 24 h. The cells on the upper side of the membrane were scraped with a cotton swab, and the cells on the bottom side of the membrane were stained with 4′,6-diamidino-2-phenylindole (DAPI). The membranes were washed with PBS and photographed after drying. The top chambers were coated with Matrigel before 2.5 × 10^4^ cells were plated for the Matrigel invasion assay. Images were taken after 72 h.

### Real-time quantitative PCR (qRT-PCR)

Total RNA was extracted from the indicated cells using TRIzol Reagent (Invitrogen, Carlsbad, CA). Total RNA concentration was determined using a NanoDrop ND-1000 spectrophotometer. The PrimeScript 1st Strand cDNA Synthesis Kit (Takara, Shiga, Japan) was used to synthesize complementary DNA (cDNA) following the manufacturer’s instructions. Quantitative RT‒PCR (qRT‒PCR) was performed using PowerUp SYBR Green Master Mix (ThermoFisher Scientific, Waltham, MA). The amplification conditions were as follows: 94 °C for 3 min; 40 cycles of 95 °C for 20 s, 60 °C for 40 s, and 72 °C for 20 s; and elongation at 72 °C for 5 min. The mRNA expression level of target genes was normalized to GAPDH using the 2-ΔΔCt method. Sangon Biotech synthesized the PCR primers used in this study (Sangon Biotech, Shanghai, China). Moreover, the sequences are shown in Supplementary Table 2.

### Western blotting

Western blotting was performed as described previously [51]. Total proteins were extracted from the indicated cells with RIPA lysis buffer (Invitrogen). The protein concentration was measured using the BCA protein assay kit (Beyotime, Shanghai, China). Western blotting was performed using a sodium dodecyl sulfate‒polyacrylamide gel electrophoresis (SDS‒PAGE) system. The PVDF membranes were blocked with 5% nonfat milk for 1.5 h at room temperature and then incubated overnight with the corresponding primary antibodies at 4 °C. Subsequently, the PVDF membranes were washed with TBST and incubated with secondary antibodies. The corresponding band was visualized with BeyoECL Plus reagent (Beyotime). The primary antibodies used for Western blotting are listed in Supplementary Table 3.

### Coimmunoprecipitation (CO-IP)

Cell protein extracts were isolated using Pierce IP Lysis Buffer (Thermo Fisher Scientific). Then, cell lysates were incubated with 5 μg of specific antibody at 4 °C overnight. Dynabeads Protein G beads (Thermo Fisher Scientific) were incubated with the obtained protein complex for 6 h at 4 °C. The magnetic bead-Ab-Ag complex was washed three times with IP buffer, eluted with loading buffer, heated for 10 min at 100 °C, and resolved by sodium dodecyl sulfate-polyacrylamide gel electrophoresis (SDS‒PAGE), followed by mass spectrometry or immunoblotting.

### Immunohistochemistry (IHC) staining

Tissue sections were deparaffinized in xylene and rehydrated with ethanol, and blocking of endogenous peroxidase activity with 3% hydrogen peroxide was followed by microwaving in 0.01 M sodium citrate buffer for antigen retrieval, after which the slides were preincubated in 10% normal goat serum for 1 h, followed by incubation overnight at 4 °C with the indicated antibodies. Afterwards, the expression of the indicated proteins was detected by a horseradish peroxidase detection system according to the manufacturer’s instructions (DAKO, Glostrup, Denmark).

The immunohistochemically stained tissue sections were scored separately by three pathologists blinded to the clinicopathological parameters. The staining intensity was scored as follows: 0, no staining (negative); 1, yellow (weak); 2, brownish yellow (medium); and 3, brown (strong). In the same tissue, multiple high-power fields with different staining intensities were viewed, and the percentage of cholangiocarcinoma-positive cells was calculated separately and then taken as the average. Scores for staining intensity and percentage of positive cells were then multiplied to generate the immunoreactivity score for each case. Samples with a final staining score of ≤ 1 were considered to have low expression, and those with a score of >1 were considered to have high expression.

### Immunofluorescence (IF)

For immunofluorescence staining, sections of formalin-fixed, paraffin-embedded tissue samples or cells fixed with cold methanol were costained with the indicated antibodies against TBK1 and then incubated with the appropriate secondary antibodies (Thermo Fisher) labelled with either Alexa 488 or Alexa 555 according to the manufacturer’s instructions. DAPI (Thermo Fisher) was used to label the nuclei.

### Constructs, transfection, and reagents

We used the wild-type TBK1 plasmid purchased from Jikai company as a template and applied mutagenesis primers to amplify the CDS of S172A mutated TBK1 using the Mut Express II Fast Mutagenesis Kit V2 (VAZYME, C214-02) according to the manufacturer’s instructions. The S172A mutant TBK1 recombinant lentiviral plasmid was constructed by double digestion with XbaI (Thermo Scientific, FD0684) and NotI (Thermo Scientific, FD0593) and ligated to the pCDH-CMV-GFP plasmid with T4 ligase (Thermo Scientific, EL0011). Lentiviral particles containing shTBK1 were obtained from GeneChem. Specific siRNAs targeting TBK1 were from Ribobio (Guangzhou, China). Cells were transiently transfected with Lipofectamine RNAiMAX transfection reagent for plasmid transfection (Invitrogen). For siRNA and HDO transfection, cells were transiently transfected with Lipofectamine 3000. The sequences are shown in Supplementary Table 2.

### Gelatin degradation assay

CCA cells were seeded onto FITC-gelatin (Thermo Scientific) substrates in a 12-well plate. After 24 h of culture, cells were fixed with methanol and TBK1 was stained through immunefluorescence. Finally, images were acquired using a fluorescence microscope.

### Design and synthesis of cholesterol-modified HDOs

Cho-TBK1-HDO designed to target TBK1 mRNA were synthesized by Tsingke Biotechnology (China). Detailed sequence information is shown in Supplementary Table 2. FAM fluorophores were covalently bound to the 5′ ends of ASO. To generate Cho-TBK1-HDO, equimolar concentrations of ASO and cRNA strands were heated in PBS at 95 °C for 5 min and then cooled to room temperature over 1 h. For in vivo studies, 0.75 mg/kg Cho-TBK1-HDO were administered to orthotopic CCA mice via tail vein injection, and the injections were repeated once a week for 3 weeks. OCT-embedded frozen liver sections containing FAM-labelled Cho-TBK1-HDO were used for IF staining.

### In vivo models

For the mouse subcutaneous xenograft model, 2 × 10^6^ cells were injected subcutaneously into the flanks of male nude mice (BALB/c, 6 weeks of age, *n* = 7 per group). After 4 weeks, the mice were sacrificed, and the subcutaneous tumours were excised, imaged, and embedded in paraffin [50]. In the lung metastasis model, 2 × 10^6^ cells were injected into male nude mice through the tail vein (BALB/c, 6 weeks of age, *n* = 7 per group). After 4 weeks, the mice were sacrificed, and the lungs were excised, imaged, and embedded in paraffin. The orthotopic mouse model of CCA was established as described previously [52]. Briefly, mice were anaesthetized, and 2 × 10^6^ cells were injected into the subcapsular region of the middle lobe. After 4 weeks, the mice were sacrificed, and the livers were excised, imaged, and embedded in paraffin. Tumour metastases were confirmed by haematoxylin and eosin staining.

All rats and mice were purchased from Guangdong Medical Laboratory Animal Center. The Jinan University Laboratory Animal Ethics Committee reviewed and approved the experimental animal protocol (ethics approval number: 20220222-40). This study conformed to the Guide for the Care and Use of Laboratory Animals published by the US National Institutes of Health. All rats and mice were housed under specific pathogen-free (SPF) conditions at the animal experimental centre of the Experimental Animal Center of Jinan University.

### Statistical analysis

Student’s *t*-test or the Mann‒Whitney U test was used to compare subgroup values, and variance analysis (ANOVA) was performed for analysis among multiple groups. Pearson’s chi-squared test was used to evaluate the correlation. The Kaplan–Meier method was used to determine survival probability, and the log-rank test was used to assess the differences. Data are expressed as the mean ± standard deviation (SD) of at least three biological replicates. The threshold for statistical significance was set at *P* < 0.05. All analyses were performed using SPSS software (Version 23.0, IBM, Armonk, NY).

## Supplementary information

Supplemental Material

Supplementary Figure 1

Supplementary Figure 2

Supplementary Figure 3

Supplementary Figure 4

Supplementary Figure 5

Supplementary Figure 6

Supplementary Figure 7

Supplementary Figure 8

Supplementary Figure 9

Supplementary Figure 10

Supplementary Figure 11

Supplementary table 1

Supplementary table 2

Supplementary table 3

## Data Availability

The datasets used and/or analyzed during the current study are available from the corresponding authors (hongjian7@jnu.edu.cn) upon reasonable request.
