# Supplementary material for: Serine/threonine kinase TBK1 promotes cholangiocarcinoma progression via direct regulation of β-catenin
Source: Oncogene. 2023 Mar 16;42(18):1492–507. doi: 10.1038/s41388-023-02651-4 (PMC10154201; doi:10.1038/s41388-023-02651-4)
Supplement: Supplementary file 1 — Supplemental Material [file 41388_2023_2651_MOESM1_ESM.doc]

**Supplementary Information**

**Serine/threonine kinase TBK1 promotes cholangiocarcinoma progression via direct regulation of β-catenin**

Chong-Qing Gao^1*^, Zhen-Zhen Chu^1*^, Di Zhang^1*^, Yang Xiao^2^, Xing-Yan Zhou^3^, Jun-Ru Wu^1^, Hui Yuan^4^, Yu-Chuan Jiang^2^, Dong Chen^5^, Ji-Chun Zhang^6^, Nan Yao^1#^, Kai-Yun Chen^7#^ and Jian Hong^1,2#^

^1^Department of Pathophysiology, School of Medicine, Jinan University, Guangzhou, Guangdong 510630, China

^2^Department of Hepatological Surgery, the First Affiliated Hospital, Jinan University, Guangzhou, Guangdong 510632, China

^3^School of Medicine, Jinan University, Guangzhou, Guangdong 510632, China

^4^Department of Gastroenterology, Huizhou Municipal Central Hospital, Huizhou 516001, Guangdong, China.

^5^Center of Hepato⁃Pancreato⁃Biliary Surgery, the First Affiliated Hospital, Sun Yat⁃sen University, Guangzhou 510080, China

^6^Department of Physiology, School of Medicine, Jinan University, Guangzhou 510632, China

^7^Department of General Surgery, Guangzhou Hospital Of Integrated Traditional And West Medicine, Guangzhou, Guangdong510632, China

***These authors contributed equally to this work.**

**^#^Corresponding authors.**

**Contact Information:**

Dr. **Jian Hong**, Department of Pathophysiology, School of Medicine, Jinan University; E-mail: [hongjian7@jnu.edu.cn](mailto:hongjian7@jnu.edu.cn); Tel: +86-20-8522-0253.

Dr. **Kaiyun Chen,** Department of General Surgery, Guangzhou Hospital Of Integrated Traditional And West Medicine; E-mail: [chky3213300@126.com](mailto:chky3213300@126.com); Tel: +86-20-8522-0816.

Dr. **Nan Yao,** Department of Pathophysiology, School of Medicine, Jinan University; E-mail: [yaon107@jnu.edu.cn](mailto:yaon107@jnu.edu.cn); Tel: +86-20-8522-0936.

**Conflicts of Interest** The authors disclose no conflicts.

**Supplementary Fig 1. TBK1 is upregulated in CCA. (A)** The levels of TBK1 mRNA expression in different types of human cancer were determined using Tumor Immune Estimation Resource (TIMER). **(B)** Representative images of IHC staining for TBK1 in our cohort , including negative (−, 2.7%); weakly positive (+, 20.9%); moderately positive (++, 45.6%) and strongly positive (+++, 30.8%). The scale bars are 200 μm (upper) and 50 μm (lower). **(C)** Quantification of TBK1 expression in our cohort (n =182). The data are the means ± SD. The pie chart in the right panel shows the proportion of high and low TBK1 expression in the tested ICC samples. **(D)** Representative immunofluorescence images of TBK1 (red) and CK-19 (green) in human ICC tissues. Scale bar, 100 μm. NS, not significant, ***P* < 0.01, ****P* < 0.001.

**Supplementary Fig 2 TBK1 promoted CCA cell migration and invasion *in vitro.* (A**) Representative data from wound healing migration assays were performed with the TBK1 stable depletion and overexpression cells. The data are the means ± SD and represent three independent experiments. Scale bars = 400 µm. **(B)** Representative data from Transwell migration and Matrigel invasion assays were performed with the TBK1 stable depletion and overexpression cells. The data are the means ± SD and represent three independent experiments. Scale bar = 200 μm. ****P* < 0.001.

**Supplementary Fig 3. Transient depletion of TBK1 inhibits human CCA cell growth, migration, and invasion *in vitro*.** **(A)** Knockdown of TBK1 in HuCCT1 and RBE cells, As confirmed by immunoblot analysis. **(B)** The effects of TBK1 transient depletion on proliferation using Cell Counting Kit-8 assay. **(C)** Representative data from wound healing migration assays were performed with the TBK1 transient depletion cells. The data are the means ± SD and represent three independent experiments. Scale bars = 400 µm. **(D)** Representative data from Transwell migration and Matrigel invasion assays were performed with the TBK1 transient depletion cells. The data are the means ± SD and represent three independent experiments. Scale bar = 200 μm. ****P* < 0.001.

**Supplementary Fig 4. TBK1 promoted CCA cell proliferation *in vivo*. (A)** Representative images (gross, H&E, and IHC) in the xenograft tumors. Scale bars = 100 μm. **(B)** In human ICC tissues, representative images of H&E and IHC staining of TBK1, CK19, and Ki67. The data are presented as the means ± SD. Scale bars, 200 μm (upper), 100 μm (lower). ****P* < 0.001.

**Supplementary Fig 5. Changes in gene profile expression after knockdown of TBK1. (A)** Log ratio–average (M-A) plots showing the gene expression changes in HuCCT1 cells after TBK1 knockdown. **(B)** Significantly upregulated or downregulated genes in HuCCT1 cells after TBK1 knockdown were detected by RNA sequencing.

**Supplementary Fig 6. TBK1 promoted the EMT process. (A)** Heatmap of EMT-related transcription factors following TBK1 knockdown.

**Supplementary Fig 7. TBK1 pharmacological inhibition inhibits CCA cells proliferation *in vivo*. (A)** Structural formulas of the TBK1 inhibitors GSK-8612. **(B)** GSK-8612 inhibited cell proliferative effect in HuCCT1 and TFK1 cells in a dose and time-dependent manner. **(C)** Immunoblots of indicated proteins in HuCCT1 and TFK1 cells treated with different concentrations of GSK8612. ****P* < 0.001.

**Supplementary Fig 8. TBK1 promoted CCA cell growth and metastasis in vivo.** **(A, B)** Representative images of gross, H&E and IHC staining Ki67 in the xenograft tumors. Scale bars = 100 μm. **(C)** The representative images of tumor morphology of liver tissue in vivo.

**Supplementary Fig 9. LDLR is up-regulated in CCA.** **(A)** Fluorescence micrographs of various mice organs 6 h after injection of 0.75 mg/kg 6-FAM-labeled TBK1-HDO. Green: 6-FAM-labeled DNA/LNA gapmer; Red: Actin; Blue: DAPI. Scale bar = 100 μm. **(B)** Western blot analysis of LDLR expression in CCA cells.

**Supplementary Fig 10. Upregulation of TBK1 predicts poor survival in ICC patients.** **(A)** OS curves of patients from Shanghai Outdo Biotech Company (cohort 1, n=91) with high or low TBK1 expression levels. **(B, C)** Overall survival (OS) and disease-free survival (DFS) curves of patients from The Cancer Genome Atlas (TCGA) dataset with high or low TBK1 expression levels. **(D)** Univariate and multivariate analyses of factors possibly associated with overall and disease-free survival after resection in our cohort (n = 182). ***P* < 0.01, ****P* < 0.001.

**Supplementary Fig 11. TBK1 is highly expressed in cholangiocarcinoma with a mesenchymal phenotype. (A)** Heatmap of mesenchymal and non-mesenchymal molecular subtypes. **(B, C)** The mRNA level of TBK1 between mesenchymal and non-mesenchymal of CCA tissues from **(B)** GSE32225 and **(C)** GSE132035. **(D)** Heatmap of the correlation between pathway enrichment scores and TBK 1 expression values.
