# Supplementary figures and images for: Serine/threonine kinase TBK1 promotes cholangiocarcinoma progression via direct regulation of β-catenin
Source: Oncogene. 2023 Mar 16;42(18):1492–507. doi: 10.1038/s41388-023-02651-4 (PMC10154201; doi:10.1038/s41388-023-02651-4)

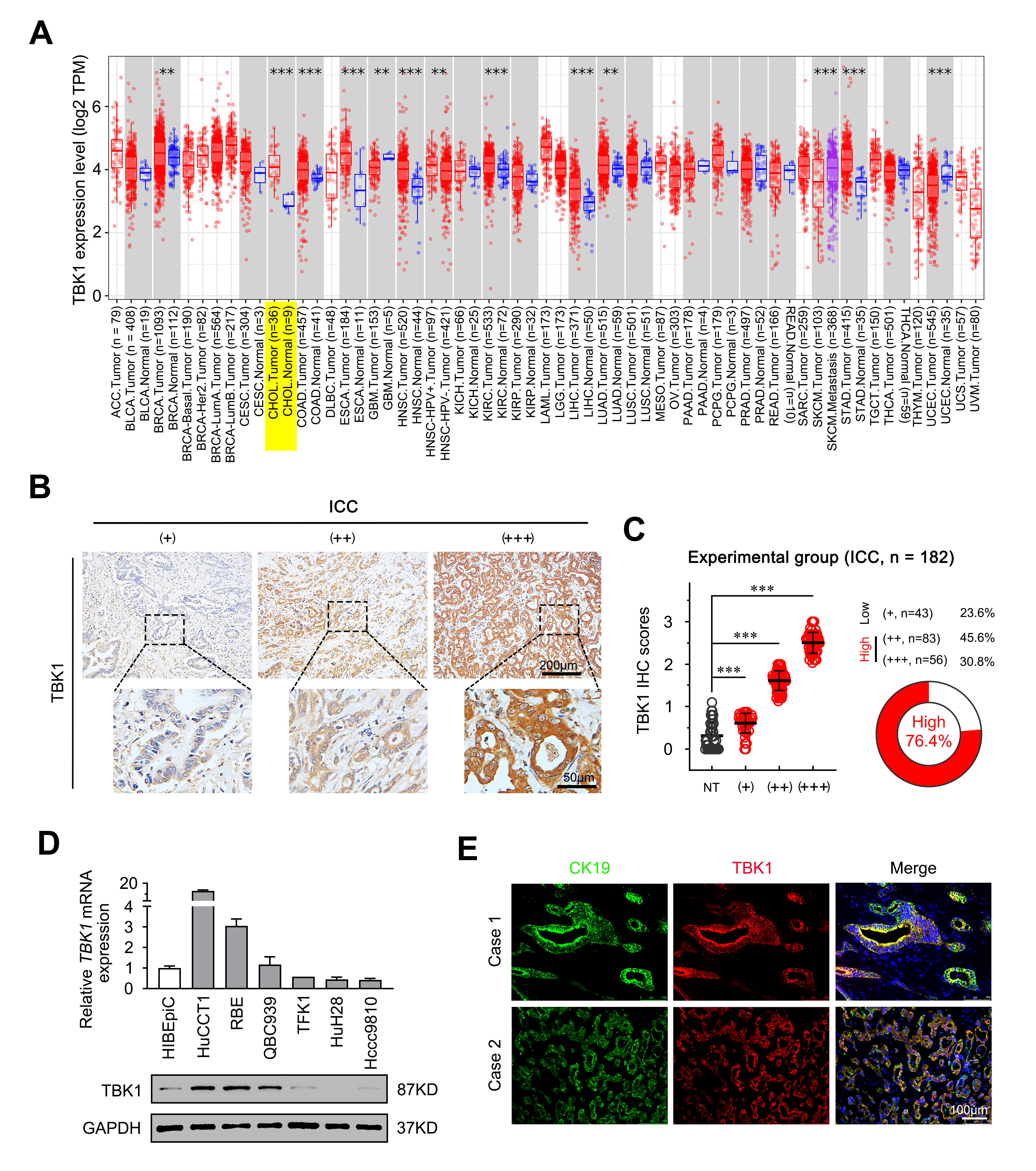

Supplement: Supplementary file 2 — Supplementary Figure 1 [file 41388_2023_2651_MOESM2_ESM.tif]

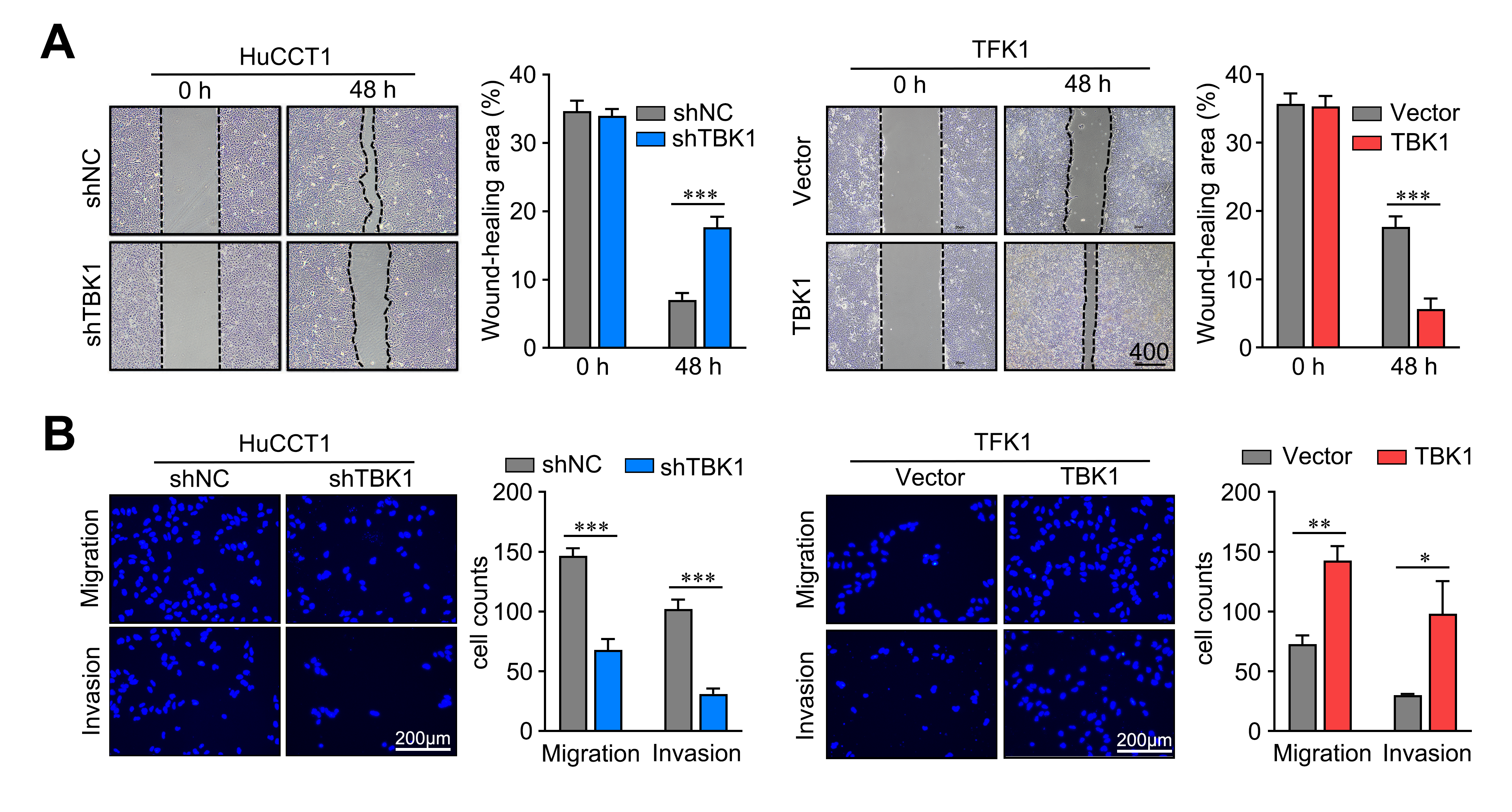

Supplement: Supplementary file 3 — Supplementary Figure 2 [file 41388_2023_2651_MOESM3_ESM.tif]

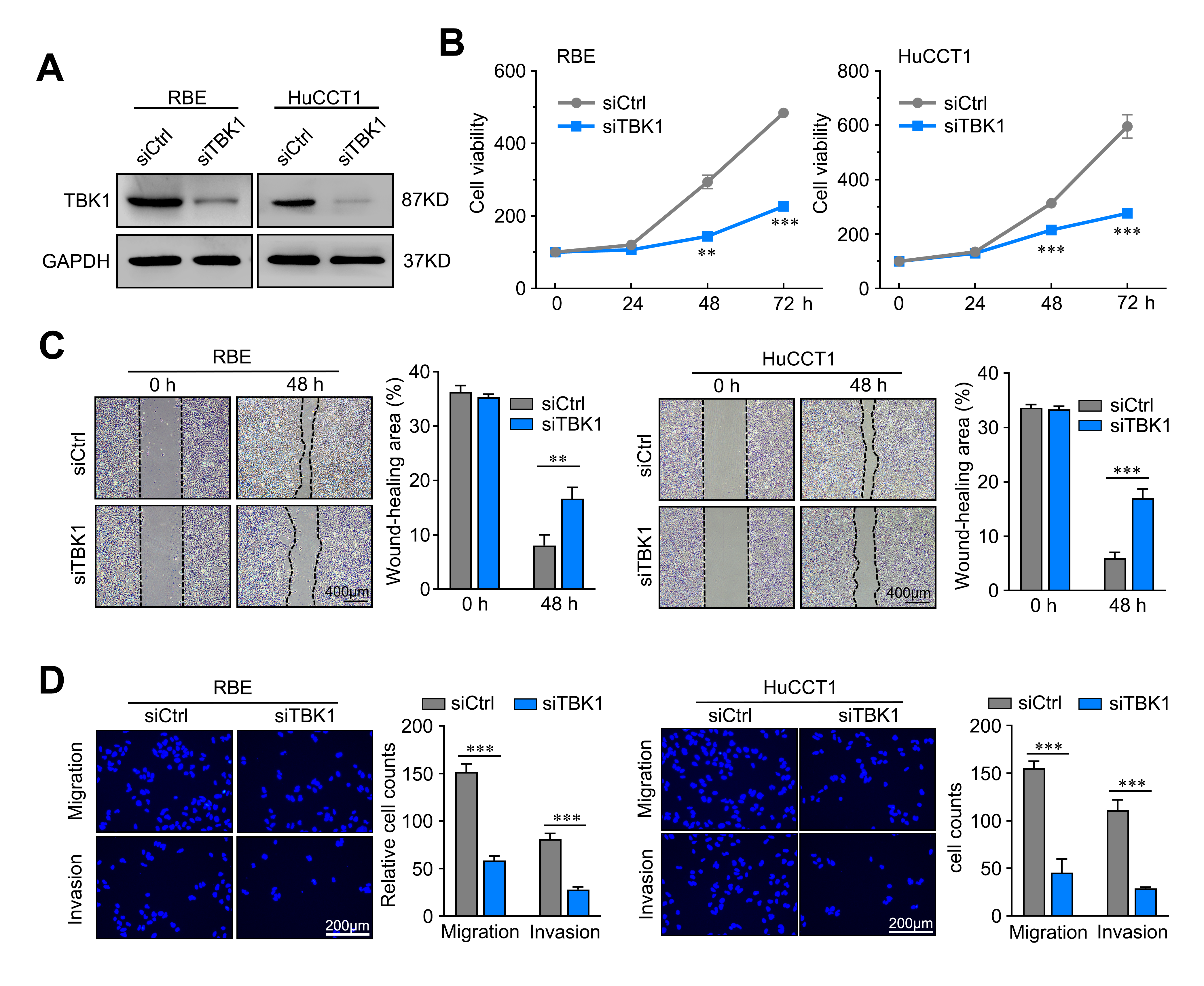

Supplement: Supplementary file 4 — Supplementary Figure 3 [file 41388_2023_2651_MOESM4_ESM.tif]

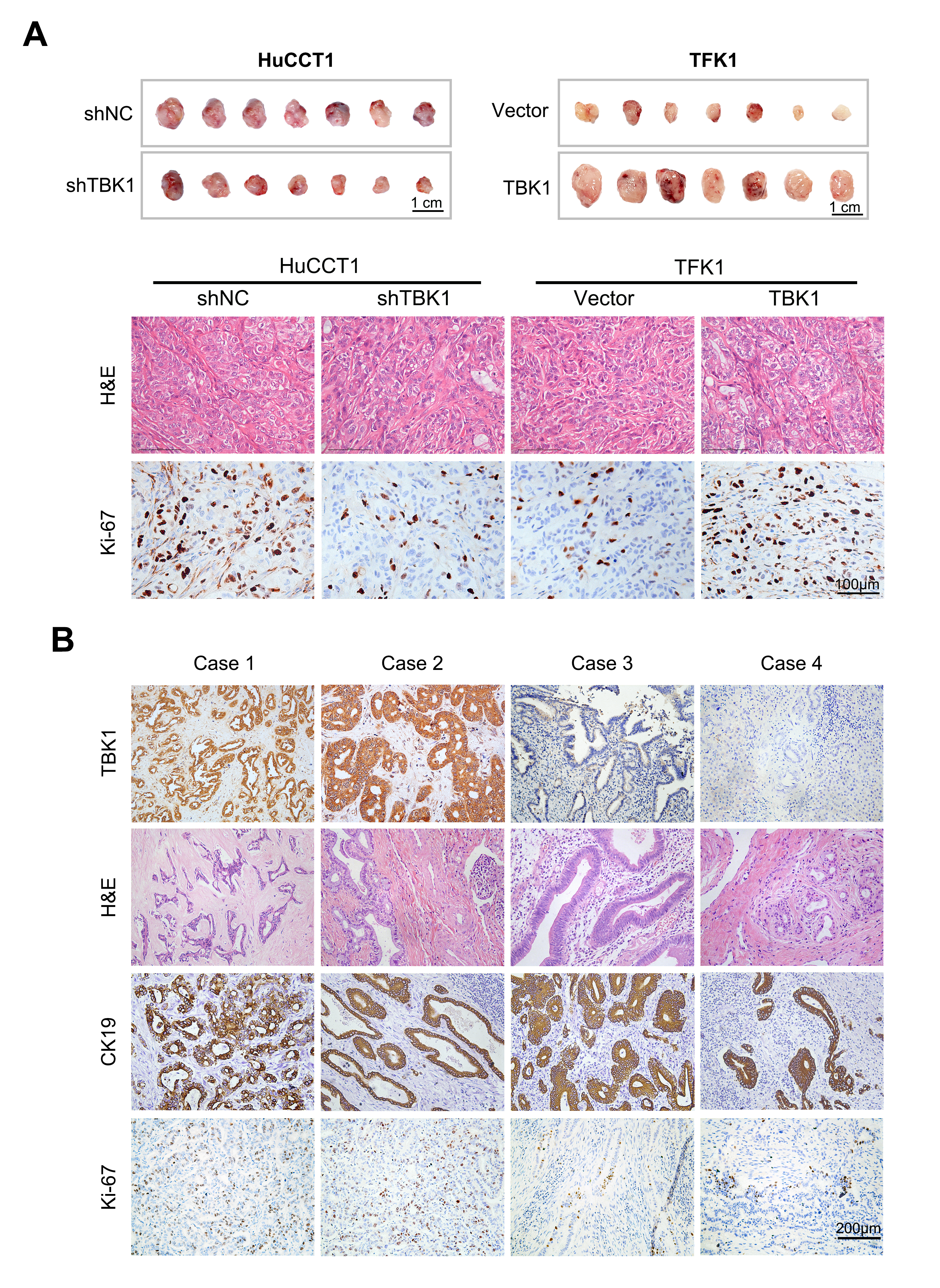

Supplement: Supplementary file 5 — Supplementary Figure 4 [file 41388_2023_2651_MOESM5_ESM.tif]

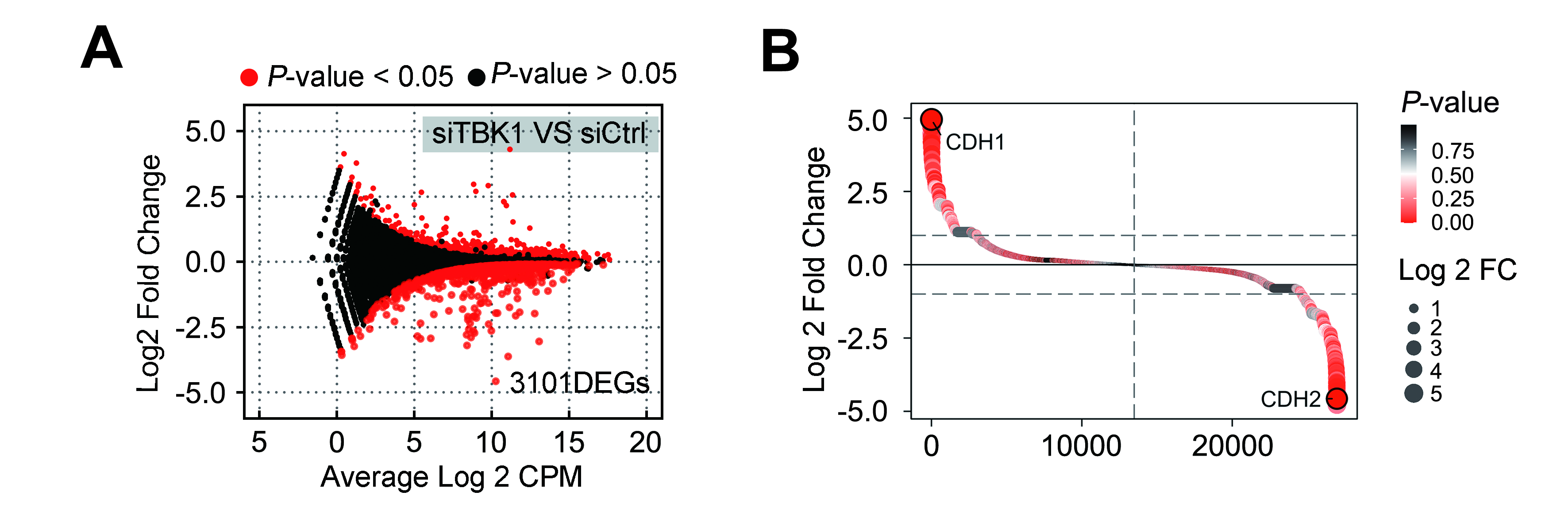

Supplement: Supplementary file 6 — Supplementary Figure 5 [file 41388_2023_2651_MOESM6_ESM.tif]

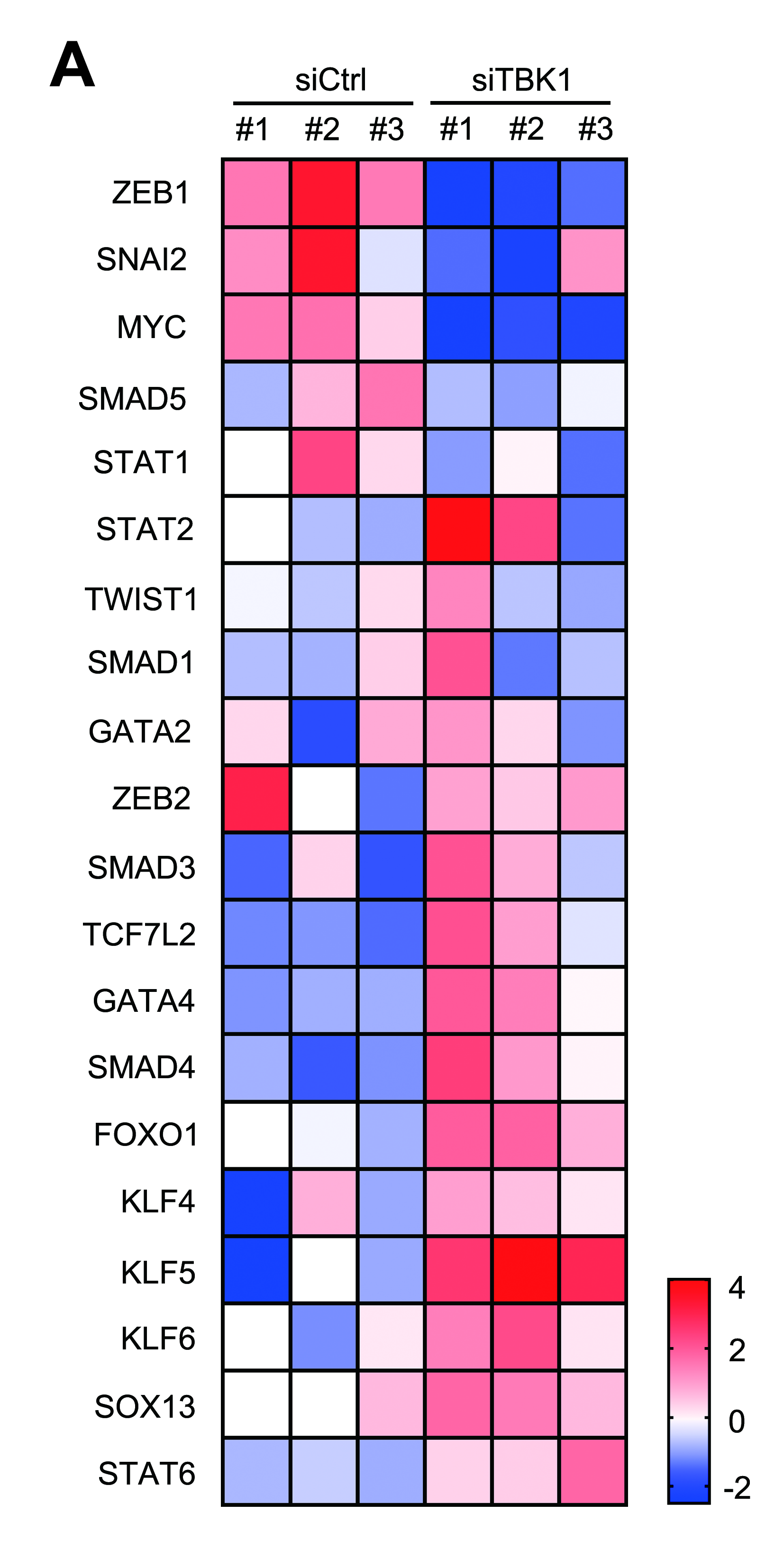

Supplement: Supplementary file 7 — Supplementary Figure 6 [file 41388_2023_2651_MOESM7_ESM.tif]

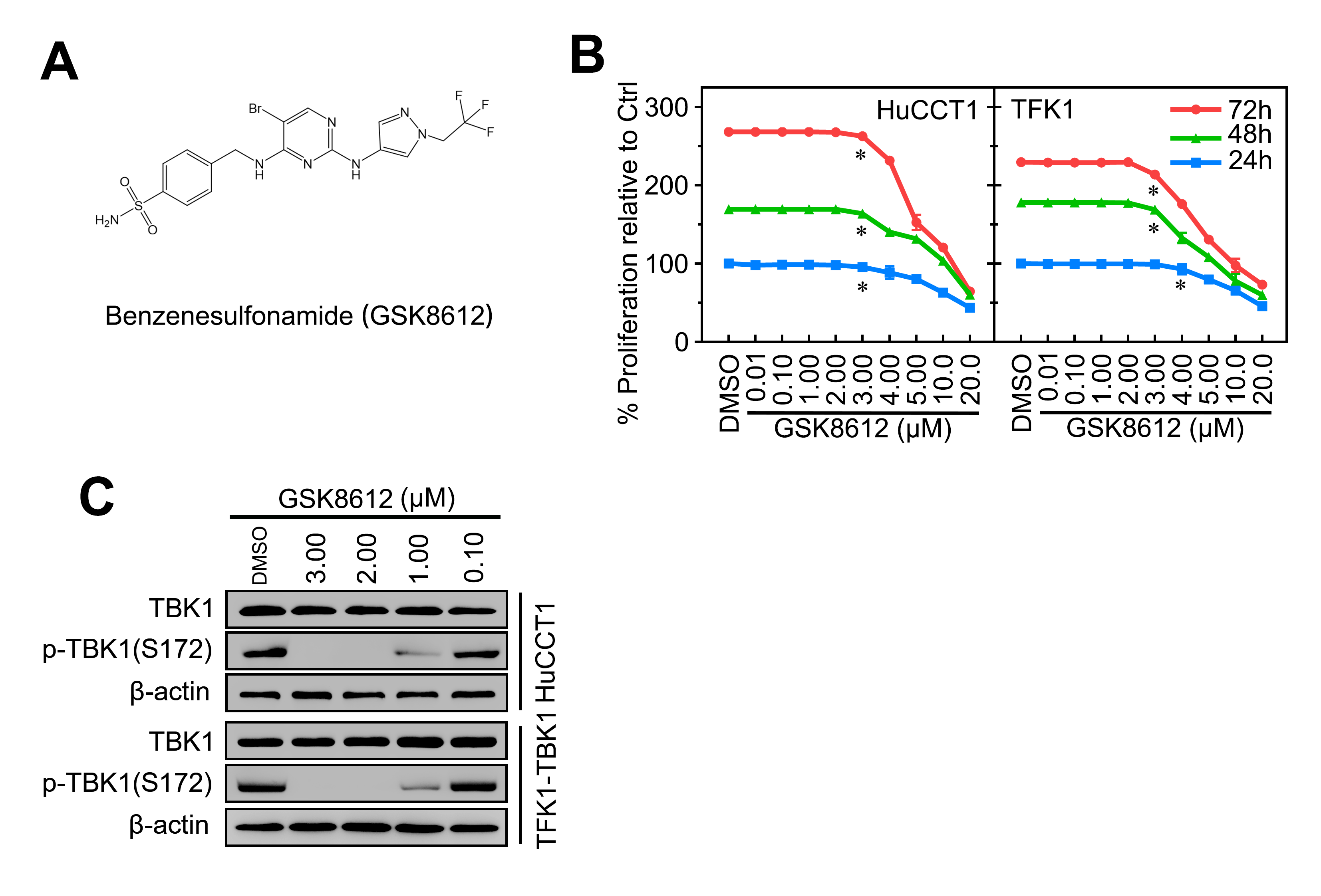

Supplement: Supplementary file 8 — Supplementary Figure 7 [file 41388_2023_2651_MOESM8_ESM.tif]

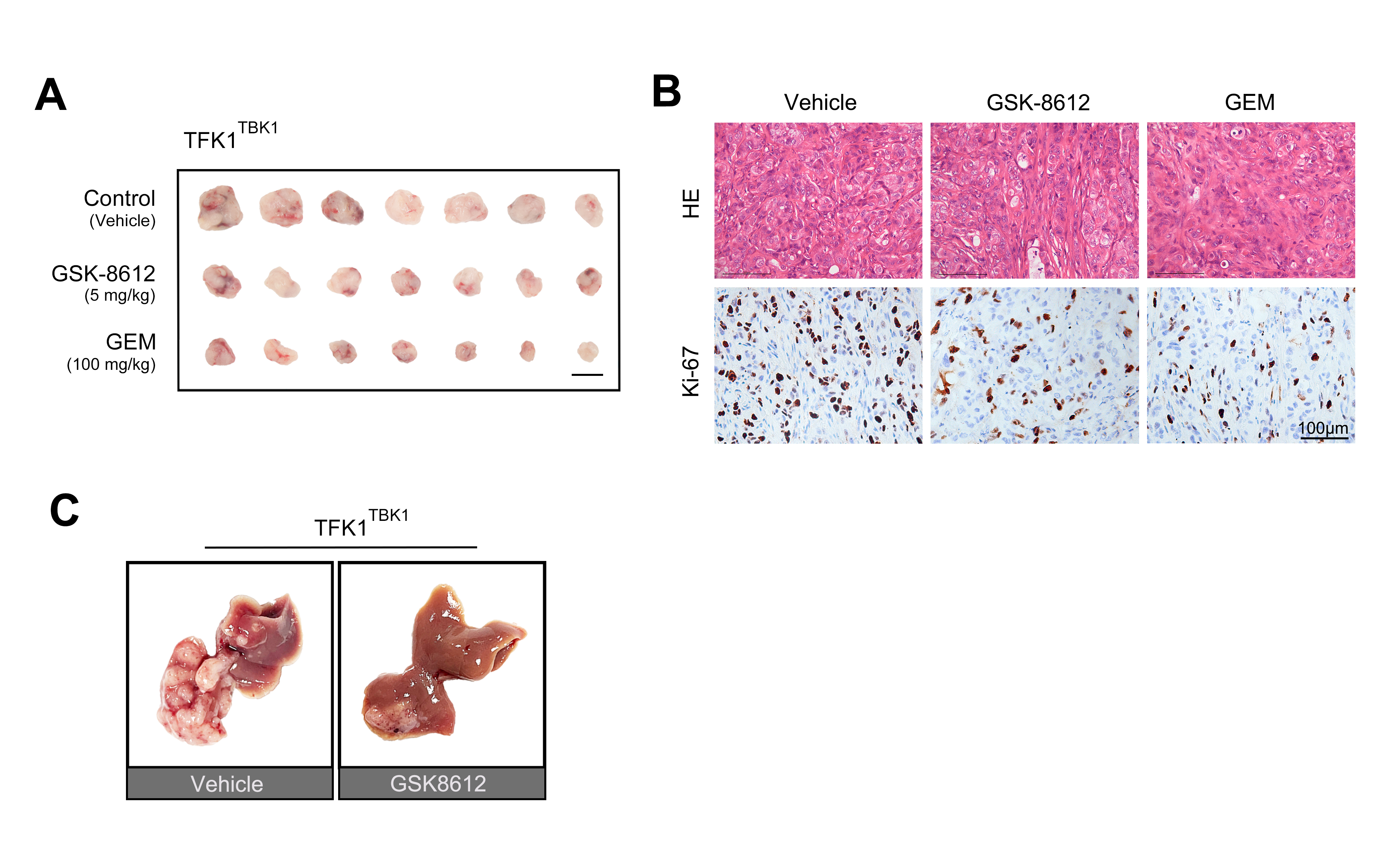

Supplement: Supplementary file 9 — Supplementary Figure 8 [file 41388_2023_2651_MOESM9_ESM.tif]

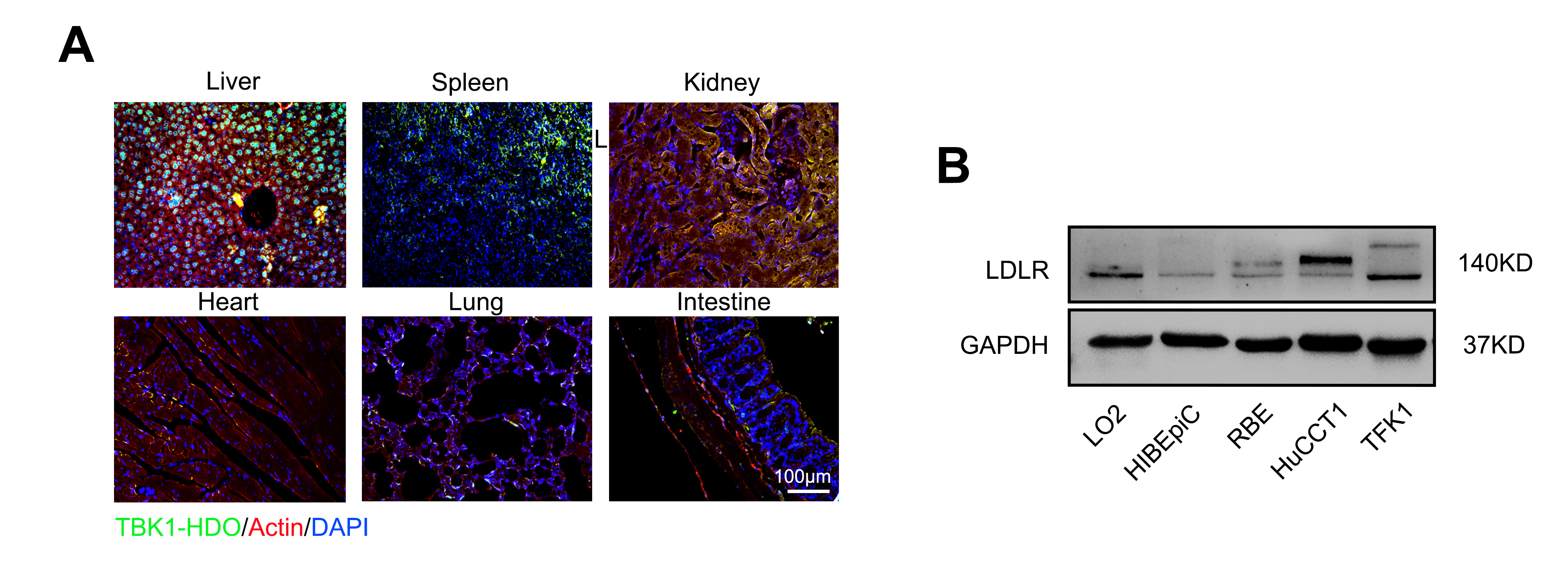

Supplement: Supplementary file 10 — Supplementary Figure 9 [file 41388_2023_2651_MOESM10_ESM.tif]

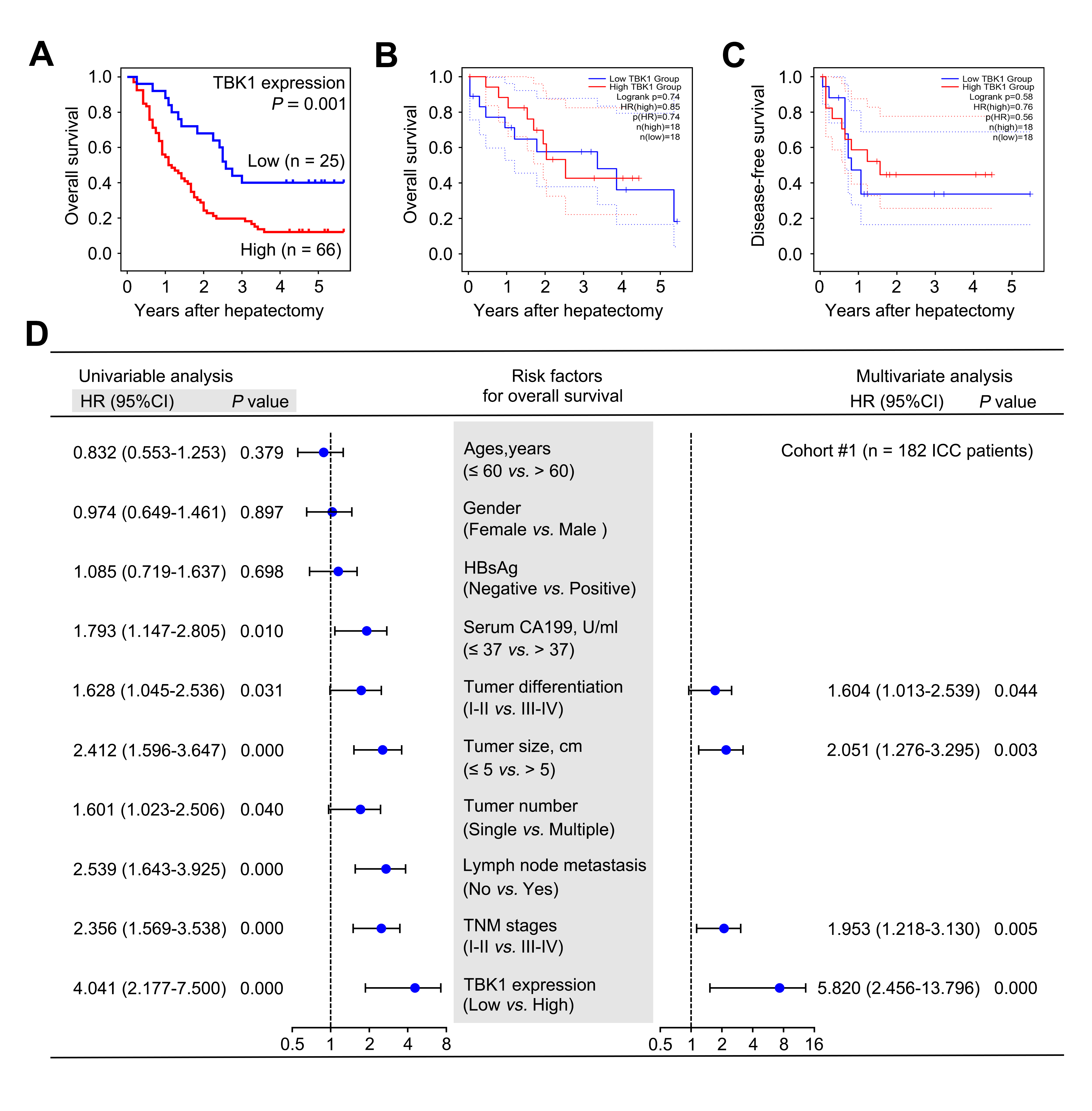

Supplement: Supplementary file 11 — Supplementary Figure 10 [file 41388_2023_2651_MOESM11_ESM.tif]

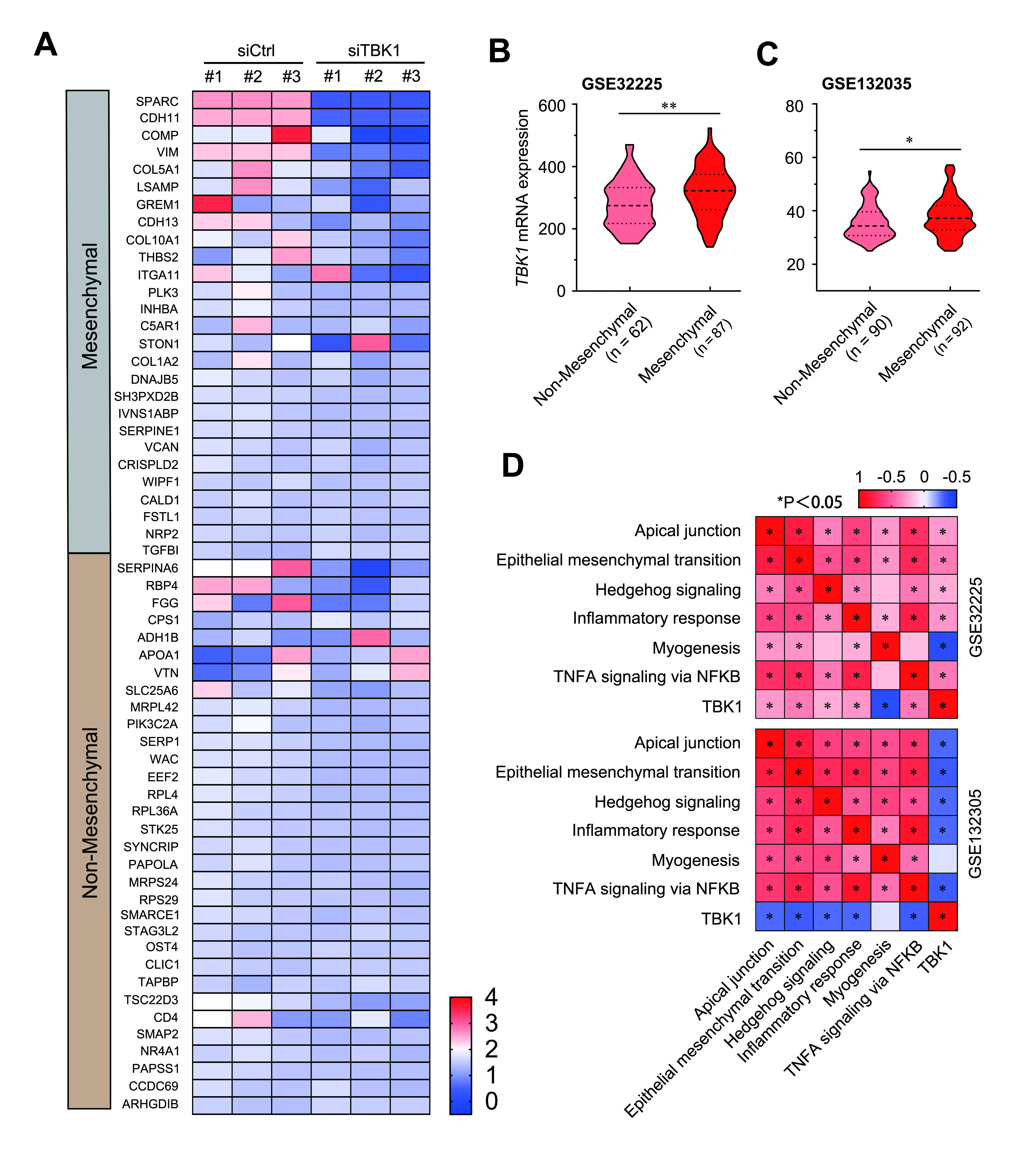

Supplement: Supplementary file 12 — Supplementary Figure 11 [file 41388_2023_2651_MOESM12_ESM.tif]
