# Supplementary material for: Serine/threonine kinase TBK1 promotes cholangiocarcinoma progression via direct regulation of β-catenin
Source: Oncogene. 2023 Mar 16;42(18):1492–507. doi: 10.1038/s41388-023-02651-4 (PMC10154201; doi:10.1038/s41388-023-02651-4)
Supplement: Supplementary file 13 — Supplementary table 1 [file 41388_2023_2651_MOESM13_ESM.doc]

**Supplementary Table 1 Correlation between TBK1 and nuclear β-catenin expression with clinicopathologic features in 182 ICC patients**

| **Characteristics** | **TBK1** | | | **β-catenin** | | |
| --- | --- | --- | --- | --- | --- | --- |
|  | **Low** | **High** | ***P* value** | **Low** | **High** | ***P* value** |
| Age (years) |  |  |  |  |  |  |
| ≤ 60 | 22 (12.1%) | 83 (45.6%) | 0.321 | 33 (18.1%) | 72 (39.6%) | 0.970 |
| **>** 60 | 21 (11.5%) | 56 (30.8%) |  | 24 (13.2%) | 53 (29.1%) |  |
| Gender |  |  |  |  |  |  |
| Male | 26 (14.3%) | 77 (42.3%) | 0.558 | 32 (17.6%) | 71 (39.0%) | 0.934 |
| Female | 17 (9.3%) | 62 (34.1%) |  | 25 (13.7%) | 54 (29.7%) |  |
| HBsAg |  |  |  |  |  |  |
| Positive | 21 (11.5%) | 51 (28.0%) | 0.155 | 22 (12.1%) | 50 (27.5%) | 0.857 |
| Negative | 22 (12.1%) | 88 (48.4%) |  | 35 (19.2%) | 75 (41.2%) |  |
| CA199, U/mL |  |  |  |  |  |  |
| ≤ 37 | 23 (12.6%) | 43 (23.6%) | **0.008*** | 26 (14.3%) | 40 (22.0%) | 0.052 |
| **>** 37 | 19 (10.4%) | 91 (50.0%) |  | 28 (15.4%) | 82 (45.1%) |  |
| NA | 1 (0.5%) | 5 (2.7%) |  | 3 (1.6%) | 3 (1.6%) |  |
| CA125, U/mL |  |  |  |  |  |  |
| ≤ 35 | 34 (19.3%) | 89 (50.6%) | 0.073 | 45 (24.7%) | 78 (42.9%) | **0.010*** |
| > 35 | 8 (4.5%) | 45 (25.6%) |  | 9 (4.9%) | 44 (24.2%) |  |
| NA | 1 (0.5%) | 5 (2.7%) |  | 3 (1.6%) | 3 (1.6%) |  |
| CEA, ng/mL |  |  |  |  |  |  |
| ≤ 37 | 33 (18.1%) | 91 (50.0%) | 0.186 | 40 (22.0%) | 84 (46.2%) | 0.484 |
| **>** 37 | 9 (4.91%) | 43 (23.6%) |  | 14 (7.7%) | 38 (20.9%) |  |
| NA | 1 (0.5%) | 5 (2.7%) |  | 3 (1.6%) | 3 (1.6%) |  |
| Tumor size (cm) |  |  |  |  |  |  |
| ≤ 5 | 32 (17.6%) | 72 (39.6%) | **0.009*** | 35 (19.2%) | 69 (37.9%) | 0.433 |
| > 5 | 11 (6.0%) | 67 (36.8%) |  | 22 (12.1%) | 56 (30.8%) |  |
| Tumor number |  |  |  |  |  |  |
| Single | 31 (17.0%) | 109 (59.9%) | 0.390 | 46 (25.3%) | 94 (51.6%) | 0.137 |
| Mutiple | 12 (6.6%) | 30 (16.5%) |  | 8 (4.4%) | 31 (17.0%) |  |
| NA | 0 | 0 |  | 3 (1.6%) | 0 |  |
| Lymph node metastasis |  |  |  |  |  |  |
| Yes | 4 (2.2%) | 54 (29.7%) | **< 0.001*** | 16 (8.8%) | 42 (23.1%) | 0.458 |
| No | 39 (21.4%) | 85 (46.7%) |  | 41 (22.5%) | 83 (45.6%) |  |
| Tumor differentiation |  |  |  |  |  |  |
| I-II | 23 (12.6%) | 79 (43.4%) | 0.871 | 33 (18.1%) | 69 (37.9%) | 0.978 |
| III-IV | 12 (6.6%) | 44 (24.2%) |  | 18 (9.9%) | 38 (20.9%) |  |
| NA | 8 (4.4%) | 16 (8.8%) |  | 6 (3.3%) | 18 (9.9%) |  |
| TNM stages |  |  |  |  |  |  |
| I-II | 33 (18.1%) | 68 (37.4%) | **0.001*** | 34 (18.7%) | 67 (36.8%) | 0.446 |
| III-IV | 10 (5.5%) | 71 (39.0%) |  | 23 (12.6%) | 58 (31.9%) |  |

**P* < 0.05 was considered to be statistically signifcant (chi-square test)

Our cohort, 182 patients from The First Affiliated Hospital Sun Yat-sen University

NA, not available
