# Supplementary material for: Serine/threonine kinase TBK1 promotes cholangiocarcinoma progression via direct regulation of β-catenin
Source: Oncogene. 2023 Mar 16;42(18):1492–507. doi: 10.1038/s41388-023-02651-4 (PMC10154201; doi:10.1038/s41388-023-02651-4)
Supplement: Supplementary file 14 — Supplementary table 2 [file 41388_2023_2651_MOESM14_ESM.doc]

**Supplementary Table 2 Sequence-Based Reagents Used in the Study**

| **Name** | **Sequence** |
| --- | --- |
| *GAPDH* human qPCR forward primer | 5’-TGCACCACCAACTGCTTAGC-3’ |
| *GAPDH* human qPCR reverse primer | 5’-GGCATGGACTGTGGTCATGAG-3’ |
| *TBK1* human qPCR forward primer | 5’-AGAACTTATCTACGAAGGGC-3’ |
| *TBK1* human qPCR reverse primer | 5’-GGATGTACTTTAGGGAGGGA-3’ |
| E-cadherin human qPCR forward primer | 5’-TCGACACCCGATTCAAAGTGG-3’ |
| E-cadherin human qPCR reverse primer | 5’-TTCCAGAAACGGAGGCCTGAT-3’ |
| Vimentin human qPCR forward primer | 5’-TGGCCGACGCCATCAACACC-3’ |
| Vimentin human qPCR reverse primer | 5’-CACCTCGACGCGGGCTTTGT-3’ |
| *Snail* human qPCR forward primer | 5’-AAGGATCTCCAGGCTCGAAAG-3’ |
| *Snail* human qPCR reverse primer | 5’-GCTTCGGATGTGCATCTTGA-3’ |
| *Twist* human qPCR forward primer | 5’-GGAGTCCGCAGTCTTACGAG-3’ |
| *Twist* human qPCR reverse primer | 5’-TCTGGAGGACCTGGTAGAGG-3’ |
| *ZEB1* human qPCR forward primer | 5’- AAGTGGCGGTAGATGGTA-3’ |
| *ZEB1* human qPCR reverse primer | 5’-TTGTAGCGACTGGATTTT-3’ |
| Human *TBK1* siRNA target sequence1 | 5’-GTACCTTACTGCTTTATCA-3’ |
| Human *TBK1* siRNA target sequence2 | 5’-GACGCACTTTACAGATGAA-3’ |
| Human *TBK1* siRNA target sequence3 | 5’-CAATGAAACTGTTCACAAA-3’ |
| Human *TBK1* shRNA target sequence1 | 5’-AGAACGTAGATTAGCTTAT-3’ |
| Human *TBK1* shRNA target sequence2 | 5’-GGCAGAGTTAGGTGAAATT-3’ |
| Human *TBK1* shRNA target sequence3 | 5’-GGAACCTCTGAATACCATA-3’ |
| *TBK1*(S172A) human PCR forward primer | 5’-GATGATGAGCAGTTTGTTGCCCTGTATGGCACAGAAGA-3’ |
| *TBK1*(S172A) human PCR reverse primer | 5’-TCTTCTGTGCCATACAGGGCAACAAACTGCTCATCATC-3’ |
| *TBK1*(HDO) target sequence | 5’-ATGTGATCACAACTTCTGTC-3’ |
