# Supplementary material for: Serine/threonine kinase TBK1 promotes cholangiocarcinoma progression via direct regulation of β-catenin
Source: Oncogene. 2023 Mar 16;42(18):1492–507. doi: 10.1038/s41388-023-02651-4 (PMC10154201; doi:10.1038/s41388-023-02651-4)
Supplement: Supplementary file 15 — Supplementary table 3 [file 41388_2023_2651_MOESM15_ESM.doc]

**Supplementary Table 3 Antibodies Used in the Study**

| **Antibodies** | **Supplier** | **Identifier** | **Working Conditions** | **Application** |
| --- | --- | --- | --- | --- |
| Anti-beta Actin | Abcam | Cat #ab8226 | 1:1000 | WB |
| Anti-Histon H3 | Abcam | Cat #ab1791 | 1:1000 | WB |
| Anti-NAK/TBK1 [EP611Y] | Abcam | Cat #ab40676 | 1:100 | IF |
|  |  |  | 1:100 | IHC |
|  |  |  | 1:1000 | WB |
| Recombinant Anti-Cytokeratin 19 [EP1850Y] | Abcam | Cat #ab7754  Cat #ab9377 | 1:400 | IF |
|  |  |  | 1:400 | IHC |
| Recombinant Anti-Ki67 [SP6] | Abcam | Cat #ab16667 | 1:100 | IHC |
| Recombinant Anti-Vimentin [EPR3776] | Abcam | Cat #ab92547 | 1:200 | IHC |
|  |  |  | 1:1000 | WB |
| Ki-67 | Abcam | Cat #ab16667 | 1:100 | IHC |
| β-Catenin | Cell Signaling Technology | Cat #8480  Cat #9562 | 1:100 | IF |
|  |  |  | 1:100 | IHC |
|  |  |  | 1:1000 | WB |
| E-cadherin | Cell Signaling Technology | Cat #3195 | 1:200 | IHC |
|  |  |  | 1:1000 | WB |
| Phospho-TBK1/NAK(Ser172) | Cell Signaling Technology | Cat #5483 | 1:1000 | WB |
| Phospho-β-catenin  (Ser33/37) | Cell Signaling Technology | Cat #2009 | 1:1000 | WB |
| Phospho-β-catenin  (Ser552) | Cell Signaling Technology | Cat #5651 | 1:1000 | WB |
| GAPDH | Cell Signaling Technology | Cat #2118 | 1:4000 | WB |
